# High-resolution mining of the SARS-CoV-2 main protease conformational space: supercomputer-driven unsupervised adaptive sampling†

**DOI:** 10.1039/d1sc00145k

**Published:** 2021-02-02

**Authors:** Théo Jaffrelot Inizan, Frédéric Célerse, Olivier Adjoua, Dina El Ahdab, Luc-Henri Jolly, Chengwen Liu, Pengyu Ren, Matthieu Montes, Nathalie Lagarde, Louis Lagardère, Pierre Monmarché, Jean-Philip Piquemal

**Affiliations:** Sorbonne Université, LCT, UMR 7616 CNRS Paris France louis.lagardere@sorbonne-universite.fr pierre.monmarche@sorbonne-universite.fr jean-philip.piquemal@sorbonne-universite.fr; Sorbonne Université, IPCM, UMR 8232 CNRS Paris France; Université Saint-Joseph de Beyrouth, UR-EGP Faculté des Sciences Lebanon; Sorbonne Université, IP2CT, FR 2622 CNRS Paris France; University of Texas at Austin, Department of Biomedical Engineering Texas USA; Laboratoire GBCM, EA 7528, CNAM, Hésam Université Paris France; Sorbonne Université, LJLL, UMR 7598 CNRS Paris France; Institut Universitaire de France Paris France

## Abstract

We provide an unsupervised adaptive sampling strategy capable of producing μs-timescale molecular dynamics (MD) simulations of large biosystems using many-body polarizable force fields (PFFs). The global exploration problem is decomposed into a set of separate MD trajectories that can be restarted within a selective process to achieve sufficient phase-space sampling. Accurate statistical properties can be obtained through reweighting. Within this highly parallel setup, the Tinker-HP package can be powered by an arbitrary large number of GPUs on supercomputers, reducing exploration time from years to days. This approach is used to tackle the urgent modeling problem of the SARS-CoV-2 Main Protease (M^pro^) producing more than 38 μs of all-atom simulations of its apo (ligand-free) dimer using the high-resolution AMOEBA PFF. The first 15.14 μs simulation (physiological pH) is compared to available non-PFF long-timescale simulation data. A detailed clustering analysis exhibits striking differences between FFs, with AMOEBA showing a richer conformational space. Focusing on key structural markers related to the oxyanion hole stability, we observe an asymmetry between protomers. One of them appears less structured resembling the experimentally inactive monomer for which a 6 μs simulation was performed as a basis for comparison. Results highlight the plasticity of the M^pro^ active site. The C-terminal end of its less structured protomer is shown to oscillate between several states, being able to interact with the other protomer, potentially modulating its activity. Active and distal site volumes are found to be larger in the most active protomer within our AMOEBA simulations compared to non-PFFs as additional cryptic pockets are uncovered. A second 17 μs AMOEBA simulation is performed with protonated His172 residues mimicking lower pH. Data show the protonation impact on the destructuring of the oxyanion loop. We finally analyze the solvation patterns around key histidine residues. The confined AMOEBA polarizable water molecules are able to explore a wide range of dipole moments, going beyond bulk values, leading to a water molecule count consistent with experimental data. Results suggest that the use of PFFs could be critical in drug discovery to accurately model the complexity of the molecular interactions structuring M^pro^.

## Abstract 2

We provide an unsupervised adaptive sampling strategy capable of producing μs-timescale molecular dynamics (MD) simulations of large biosystems using many-body polarizable force fields (PFFs).

## Graphical abstract

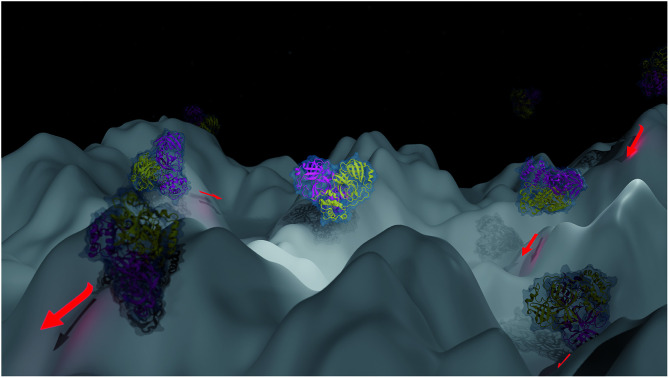

## 1 Introduction

At the end of December 2019, a novel coronavirus (CoV) that induces severe acute respiratory disease (SARS) was discovered and labeled SARS-CoV-2.^1^ It causes the disease named COVID-19, which led to a global pandemic in 2020 and finally to an urgent global issue.

Great effort has been made to gain insights into the action of the virus on the human body. As the genome of the virus has been rapidly determined,^2^ a similarity between the SARS-CoV-2 virus and the older SARS-CoV (2003) and Middle East respiratory syndrome coronavirus (MERS-CoV in 2012) was observed. Besides vaccines, researchers started the hunt for small molecules to treat the disease. Rapidly,^2^ different classes of proteins have been experimentally characterized that could be useful targets for drugs. Among the different classes of proteins that have been experimentally characterized, the main protease^3^ is essential for processing the precursor polyprotein for the replication of the virus. Indeed, proteases are responsible for activating viral proteins for particle assembly. Due to their importance within the replication cycle of the virus, they have been proven to be successful targets for antiviral agents and are used to treat many diseases including HIV and hepatitis.^4^ In the case of SARS-CoV-2, the main protease is called M^pro^ or 3CL^pro^. Many efforts have been made to refine the crystallographic structure of M^pro^ as the number of experimental structures available in the Protein Data Bank is increasing. While more than one hundred M^pro^ structures exist and massive efforts to discover a successful inhibitor are underway, computational approaches involving virtual screening and Molecular Dynamics (MD) simulations are needed to help experimentalists to *in silico* optimize their millions of test molecules.^5–8^

Molecular Dynamics is a powerful tool for understanding the structural and dynamical details of complex biological systems. It also enhances the ability to identify promising protein inhibitors. Two main research groups, DE Shaw Research (DESRES) and RIKEN Center for Biosystems Dynamics Research, recently released multi-microsecond MD simulations of the M^pro^ dimer.^5,6^ These MD conformational ensembles both used non-polarizable force fields (n-PFFs) including DES-AMBER^9^ and AMBER14ff.^10^ Although the simulations are of great help for the scientific community, conventional MD (cMD) simulation results are limited by the daunting complexity of M^pro^'s conformational space, which requires very large computational resources. In practice, both DESRES and RIKEN results were obtained on special-purpose petascale supercomputers designed for MD (Anton^11^ and MD-GRAPE-4A^12^ for DESRES and RIKEN, respectively). So, what can be done next? Besides these large scale MD simulations, the question of accuracy still remains open. Indeed, conformational space sampling depends by definition also on the force field used for the simulations. Our group has been involved for many years in the demonstration of the importance of considering explicit many-body effects in classical MD and free energy methods through the use of polarizable force fields (PFFs).^13–17^ Indeed, electronic polarization affects solvation and modifies the stability of secondary and quaternary structures of proteins, playing therefore a crucial role in defining the conformational space of a protein. Applying such methods to COVID-19 research could provide additional insights for drug modelers and experimental teams. When our project started (end of March 2020) in response to the international High-Performance Computing (HPC) global effort to mitigate the impact of the COVID-19 pandemic,^18–20^ performing long timescale MD simulations using new generations of PFFs on SARS-CoV-2 proteins encompassing hundreds of thousands of atoms (or more), such as M^pro^, was out of reach of generalist supercomputers. Such simulations would have required years of computation.

To overcome these limitations we introduce a density-driven unsupervised adaptive sampling method based on statistical models and principal component analysis (PCA). It has been deployed on a generalist supercomputer. Since the global exploration problem is decomposed into a set of separate MD trajectories, the process can be restarted using an iterative selection method, and various computations can take place on a large number of Graphics Processing Units (GPUs) that are now available in generalist supercomputers. Such a strategy enables the Tinker-HP package,^21^ which recently proposed a GPU-accelerated implementation,^22^ to perform multi-microsecond MD simulations within a few days, where years would have been required with single GPU card or CPU-based conventional MD simulations. We additionally provide the capability to re-weight our simulations, which enables full exploitation of the total amount of MD trajectories to compute statistical properties that can therefore benefit from the long simulations. After describing our sampling strategy, we will detail our conformational space exploration results that notably expand over those obtained by other groups. We will unveil critical structural behavior not fully captured with n-PFFs. We particularly investigated the differences in clustering results, active site volumes, cryptic pockets, key structural activation markers linked to the oxyanion hole structuring, interactions between the C-terminal chain and the active site, and solvation patterns of some key residues. The effect of pH is also discussed.

## 2 Unsupervised adaptive sampling strategy for exploration: exploiting pre-exascale machines and GPUs

Adaptive sampling has been used for many years and has proven to be a powerful exploration tool to study protein folding and dynamics, ligand binding and a variety of rare molecular events.^23–26^ For this family of approaches, multiple iterations of independent molecular dynamics simulations are performed, basing the initial conditions at each iteration on the results of previous iteration steps. We propose here a new unsupervised (*i.e.* fully automated) adaptive sampling strategy dedicated to our specific use of PFFs within large supercomputer systems allowing for the simultaneous use of hundreds or thousands of GPU cards. This characteristic is important as it allows us to benefit from the full potential of pre-exascale supercomputers, and will naturally transfer to future exascale machines. The results presented here benefit from a GPU acceleration in the newly developed Tinker-HP GPU code^22^ that was first used here for COVID-19 simulations. However the procedure is completely general and can be applied to any homogeneous or heterogeneous computational platforms compatible with Tinker-HP^21,27^ or any MD software. Therefore, in view of the particular distribution of available numerical resources, the simulations are organized by iterations as follows. At the beginning of each iteration, some initial structures are selected among the configurations sampled in the past iterations, from which independent MD simulations are run, generating new configurations. The selection of the initial structures at each iteration follows an adaptive procedure designed to enhance the exploration of a low-dimensional space of slow variables.

**Fig. 1:**
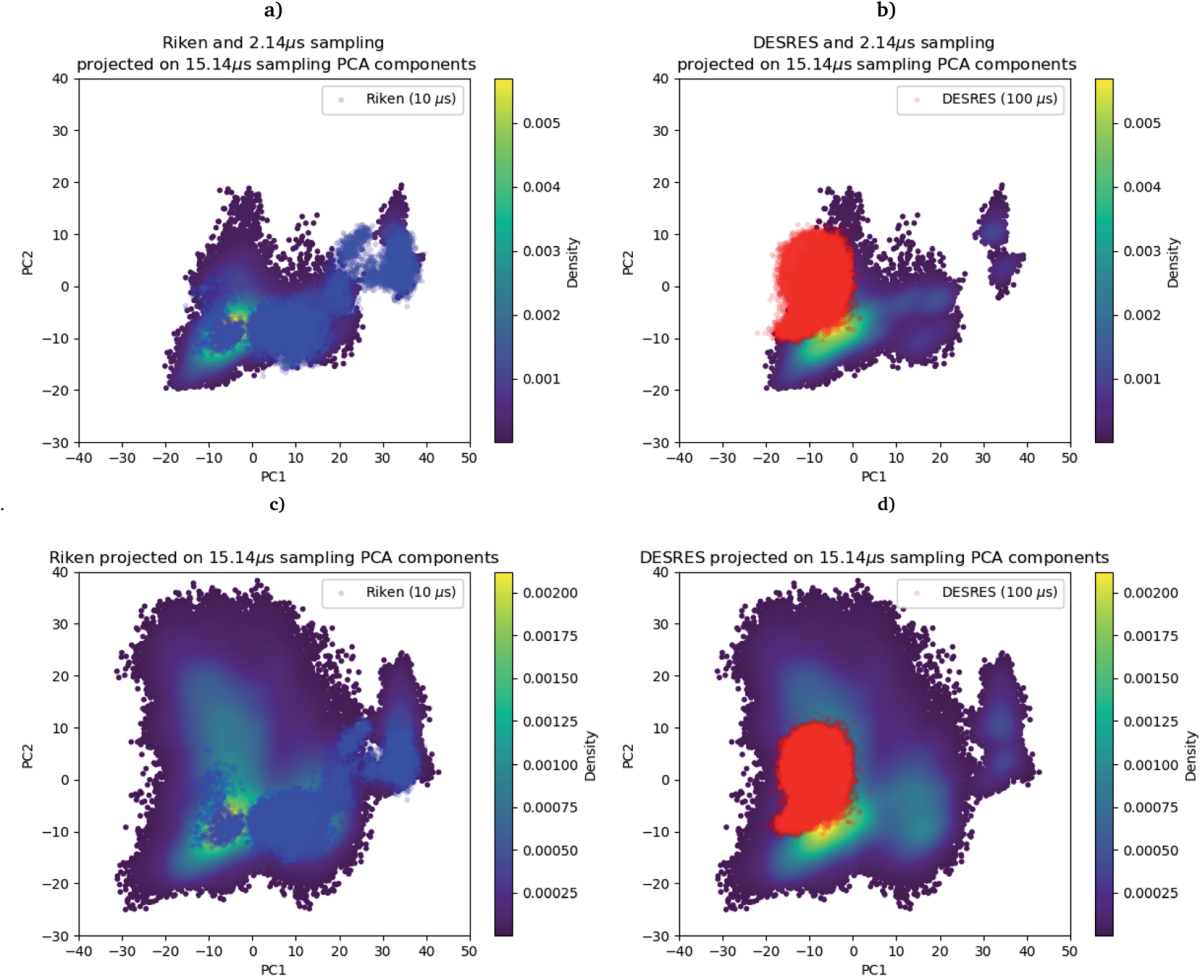
RIKEN and DESRES datasets superposed on the 6LU7 protein backbone and projected on the first two PCA components fitted to, respectively, the 2 μs (a and b) and 15.14 μs (c and d) simulations.

**Fig. 2:**
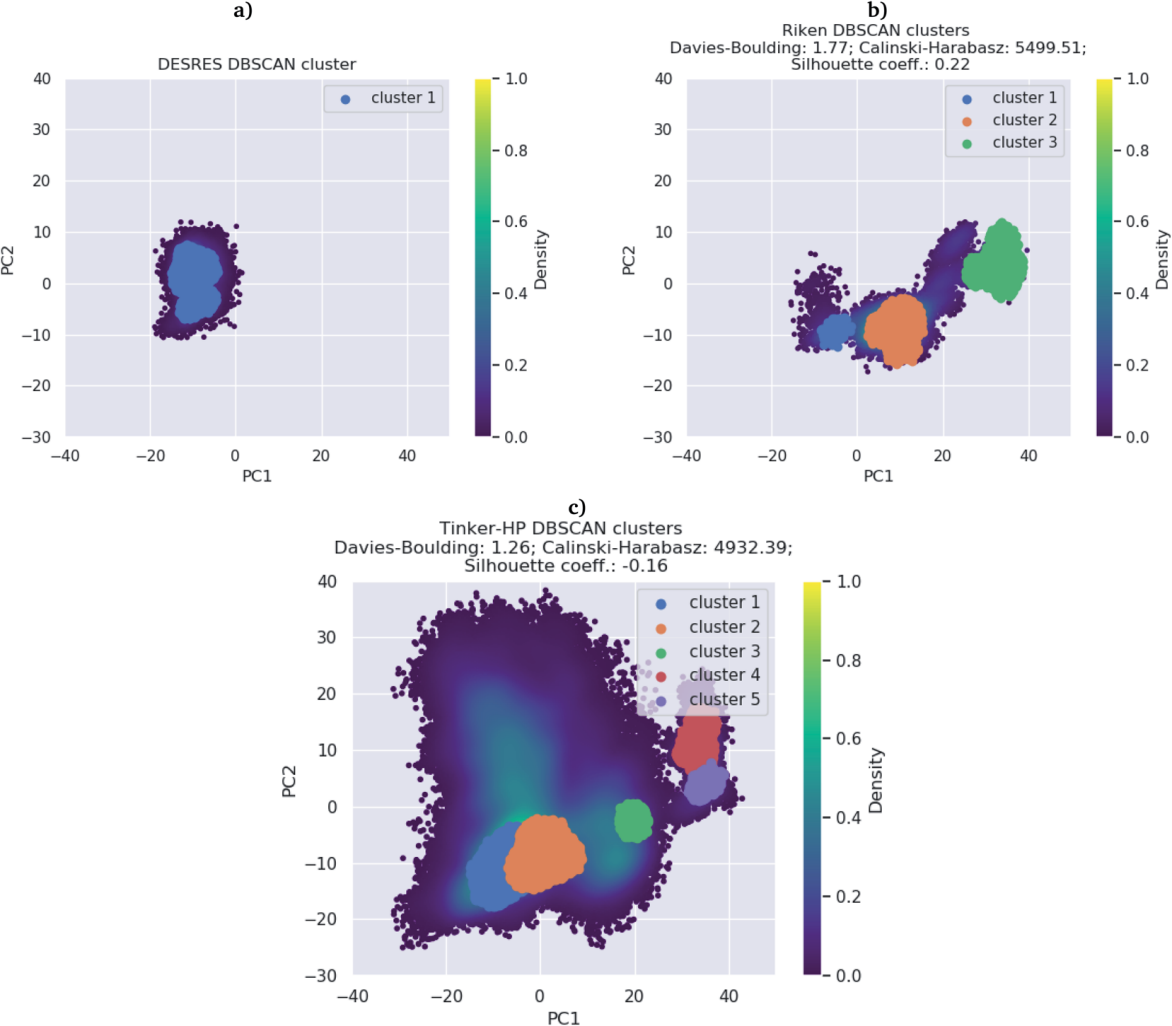
DBSCAN clustering of (a) DESRES (100 μs) and (b) RIKEN (10 μs) datasets and (c) the Tinker-HP 15 μs simulation.

More precisely, *M*_*k*_ denotes the number of configurations available at the beginning of iteration *k* ≥ 0, and (*q*_*i*_)_1≤*i*≤*M*_*k*__ the configurations. Here, a configuration means the positions 
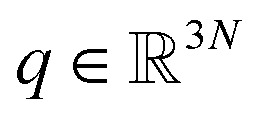
 of all the atoms of the system. In particular, at the very beginning of the algorithm, we suppose that we start with *M*_0_ ≥ 1 configurations, obtained from an initial conventional MD simulation (which is in practice non-polarizable), or previously available studies. At the beginning of iteration *k*, first, the protein is aligned in all configurations, using the backbone atoms of the 6LU7 crystal structure from the Protein Data Bank.^3^ A principal component analysis (PCA)^28^ is then performed, using the scikit-learn^29^ and MDTraj^30^ packages, on the protein atoms (*q*_*i*_)_1≤*i*≤*M*_*k*__, from which the *n* = 4 principal modes are considered. This choice was made after a global analysis of the first 20 PCA modes of the first AMOEBA 0.14 μs which showed that *n* > 4 modes had variance contributions below 4% (Fig. 1, ESI†). This has also been corroborated by an analysis of RIKEN and DESRES trajectories, for which, respectively, 3 and 4 PCA modes are above 4% (Fig. 2, ESI†). We denote by 
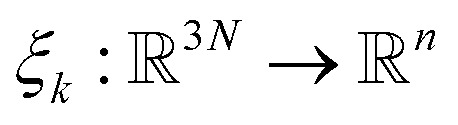
 the orthogonal projection on these *n* principal modes and we write *x*_*i*_ = *ξ*_*k*_(*q*_*i*_). At the beginning of iteration *k*, this represents the current guess of slow variables of the system, and in order to enhance the sampling, we would like to explore all the values of these slow variables. In other words, ideally, we would like the values of *x* sampled to be uniformly distributed over some compact set of 
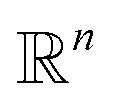
. The selection procedure is designed to push the exploration in the direction of this ideal target.

The density *ρ*_*k*_ of the collective variables is approximated by a Gaussian kernel, *i.e.* for 
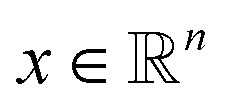

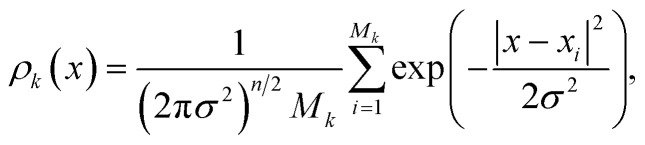
for some *σ* > 0. In practice we used the D.W. Scott method, implemented in Scipy,^31^ to estimate a suitable bandwidth *σ*. Denoted by *s*_*k*_ is the number of MD trajectories that are going to be run during iteration *k*. In order to select the initial structures 
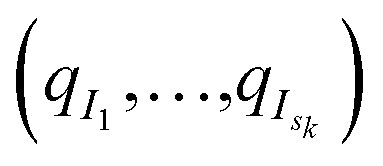
 of these simulations, the indexes *I*_1_, …, *I*_*s*_*k*__ are generated as independent random variables in {1, …, *M*_*k*_} distributed according to
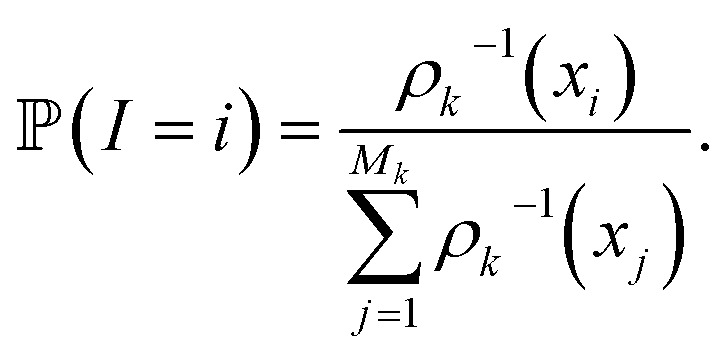


In other words, among all the structures currently available, *q*_*i*_ is selected to be the initial structure of a new simulation with a probability inversely proportional to its density (in the low-dimensional space given by the first four PCA components). The effect of this selection can intuitively be illustrated as follows: if two domains of similar size (in the sense of the Lebesgue measure on 
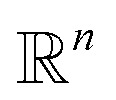
) have been visited, with one that concentrates most of the past trajectories while the other contains only a few points, then approximately half of the new initial structures will be selected in each domain; in contrast, a uniform selection among the past configurations would have put much more weight on the dense domain.

From the initial structures 
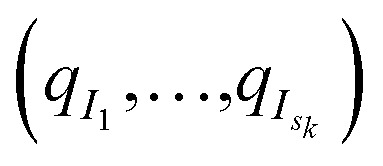
, *s*_*k*_ independent MD simulations are sampled, and the state of each simulation is recorded every 0.1 ns (the initial structure is not recorded, since it has already been recorded in one of the past iterations). Here, independent means that the initial velocities (sampled according to the equilibrium Gaussian density) and the white noises of the Langevin thermostats are independent (and, of course, independent from previous iterations, so that a trajectory starting at some configuration *q*_*i*_ will be different from the trajectory that initially produced this *q*_*i*_). At the end of this *k*th iteration, structures (*q*_*j*_)_*M*_*k*_<*j*≤*M*_*k*+1__ have been added, and iteration *k* + 1 starts.

The procedure penalizes areas that have already been extensively visited, and is in a way reminiscent of the metadynamics^32^ method except that the statistical biasing is done through a selection step between each iteration rather than a biasing force updated along the trajectory. By comparison with metadynamics, this unsupervised selection step has the advantage of overcoming the critical choice of initial collective variable at the beginning of the simulation reinforcing automation of the sampling scheme.

This strategy belongs to the family of counts based adaptive sampling algorithms, where one only exploits the number of passages in the different states (micro or macro) visited in the previous iterations to choose which state to restart trajectories from. These are known to be efficient for pure exploration purposes (as is the case here), even though more refined algorithms exist when some information is available as to where the sampling should be guided.^24^ However, in contrast to what is usually done in the context of Markov State Models (MSMs),^23^ the states are not defined by applying a clustering algorithm to the already explored structures, but are the projection on the *n* principal components generated by PCA (here, *n* = 4 as we discussed) of all the previous data. This has the advantage of providing an unsupervised sampling strategy that does not rely on a particular clustering algorithm (and therefore its associated parameters) and treating every point of this 4-dimensional representation differently.

At the end of the simulation, *M*_*K*_ configurations have been sampled with *K*, the total number of iterations. For a large *K*, the distribution of these configurations does not converge to the canonical distribution because of the statistical bias induced by the selection. To compute thermodynamic quantities, this bias should be taken into account. In that case, we interpret the previous selection as an importance sampling scheme. Thus, we have to compute a score *ω*_*i*_ > 0 for each *i* ∈ {1, …, *M*_*K*_} so that the canonical average of an observable *φ* is estimated by
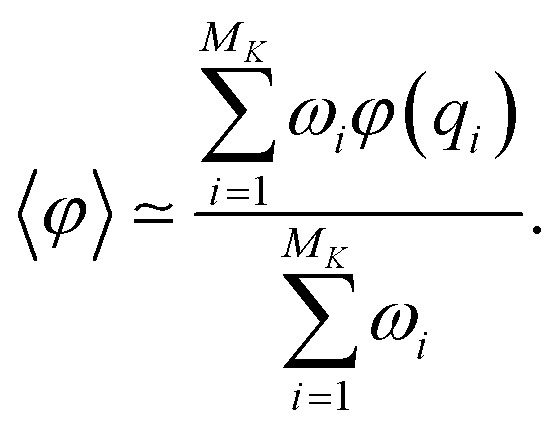


The score *ω*_*i*_ is the ratio between the probabilities to obtain *q*_*i*_ in the biased simulation and in an unbiased simulation (where, between each iteration, the next initial conditions are uniformly chosen among all currently available configurations, *i.e.* all with probability 1/*M*_*k*_). As a consequence, it is computed as follows: for all *i* ≤ *M*_0_, *ω*_*i*_ = 1. Suppose by induction that *ω*_*i*_ has been computed for all *i* ≤ *M*_*k*−1_ for some *k*. Let (*i*_1_, …, *i*_*s*_*k*__) be the indexes that have been randomly selected for the initial conditions at the beginning of iteration *k*. For each *h* ∈ {*i*_1_, …, *i*_*s*_*k*__}, *α*_*h*_ is computed:
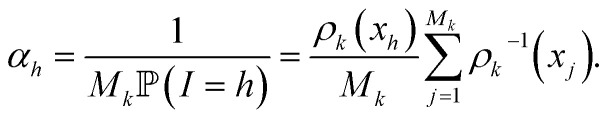


Then, the score of all the configurations that are generated during iteration *k* from the initial condition *q*_*h*_ is *α*_*h*_*ω*_*h*_. That way, *ω*_*i*_ is computed for all *i* ≤ *M*_*k*_.

This latest point is important since it means that the total simulation time can be used to compute average statistical properties that are unbiased and therefore exploitable. For example, it is possible to compare them to those obtained upon performing conventional MD runs.

Finally, it should be noticed that, instead of the PCA, this adaptive sampling strategy may be used with any other collective variables and/or dimensionality reduction algorithm. Overall the procedure is fully unsupervised, fast and can be used within Tinker-HP in a fully automated way.

## 3 Large scale unsupervised adaptive simulation using polarizable force fields (PFFs) and GPUs

### 3.1 Preparation of systems and choice of initial structures

In order to perform a large scale unsupervised adaptive sampling simulation, starting structures have to be selected from a conventional MD simulation (using either n-PFF or PFF approaches). We chose the RIKEN dataset as the starting point. From their 10 μs conventional MD simulation (PDB: 6LU7, pH = 8)^3^ using the n-PFF AMBER14ff^10^ approach and using PCA as a guiding thread, we carefully extracted 14 relevant structures that represent our starting point for the study. It is worth noting that the 6LU7 crystal structure is a holo structure including a covalently bound inhibitor. The inhibitor-unbound apo structure was initially obtained by RIKEN removing the inhibitor and relaxed over 10 μs of simulation (https://data.mendeley.com/datasets/vpps4vhryg/1). Each Amber14ff structure was then minimized with the AMOEBA PFF^33–36^ and an L-BFGS algorithm until a Root Mean Square (RMS) of 1 kcal mol^−1^ on the gradient was reached. It is important to note that not all histidine residues are protonated in the RIKEN structure similarly to the DESRES one. Since it has been recently demonstrated that the highest p*K*_a_ for possible protonation of histidine sites was lower in the SARS-CoV-2 M^pro^ than in the SARS-CoV-1 M^pro^, being about 6.6,^37^ the present simulation is therefore consistent with physiological pH conditions (pH = 7.4).^38^

### 3.2 Simulation protocol

The presented all-atom simulation was performed using the newly developed GPU module^22^ within the Tinker-HP package,^21^ which is part of the Tinker 8 platform.^39^ This newly developed module is able to efficiently exploit mixed precision^22^ offering a strong acceleration of simulations using GPUs. The 98 694 atom initial structure of the fully solvated M^pro^ dimer was extracted from the Protein Data Bank (PDB: 6LU7) and the AMOEBA PFF^33,34,36^ was used to describe all atoms (protein and water). Periodic boundary conditions using a cubic box with side lengths of 100 Å were used. Langevin molecular dynamics simulations were performed using the BAOAB-RESPA1 integrator^40^ using a 10 fs outer timestep, a preconditioned conjugate gradient polarization solver (with a 10^−5^ convergence threshold), hydrogen-mass repartitioning (HMR) and random initial velocities. Periodic boundary conditions (PBCs) were employed using the Smooth Particle Mesh Ewald (SPME) method with a grid of dimensions 128 Å × 128 Å × 128 Å. The Ewald-cutoff was taken to be 7 Å and the van der Waals cutoff to be 9 Å. As we explained, we started the simulation by running a 10 ns cMD for each of RIKEN's 14 representative structures (as mentioned in Section 3.1). A first adaptive sampling selection was then conducted on those 140 ns initial structures. We chose to use the first four PCA components (see the method section) as conformational space for the adaptive sampling method. At each iteration, the adaptive sampling procedure is then used on these newly computed first four PCA components in order to select 100 structures. Then, 100 independent molecular simulations of 10 ns were performed in the NVT ensemble at 300 K on single NVIDIA V100 GPU cards. Each trajectory belonging to the same adaptive sampling iteration was run simultaneously on the HPE Jean Zay Supercomputer (IDRIS, GENCI, France). A single adaptive sampling iteration took less than 18 hours to complete, allowing a production rate of 15.14 μs in two weeks. Overall, the simulations ran over 12 working days in line with computer center resources availability.

The complete 15.14 μs trajectories with and without water are freely accessible through the Swiss National Supercomputing Center (CSCS)^41^ and have been linked to the BioExcel/Molssi COVID-19 community portal. A movie depicting the progress of the exploration can be found in the ESI.†

### 3.3 Performance of the adaptive sampling exploration: comparisons with other available simulations

As we mentioned in the method section, we use the PCA^28^ as an intermediate quantity to orient the consecutive sampling iteration. However, it is also a good quantity to quickly assess the performance of the adaptive sampling scheme for the exploration of the conformational space. Indeed, the analysis of MD trajectories with PCA is a well-known strategy known in the community as the “essential dynamics”.^42–44^ PCA, being a dimensionality reduction algorithm that evaluates directions maximizing the variance of the dataset, is thus a revealer of a system conformational diversity. Therefore, it can be seen as a way to assess the amount of sampling and can also detect explicit “essential motions” otherwise not discernible using predefined collective variables. Thus, it is interesting to compare the amount of sampling on the space of these reduced variables. This is why we projected the RIKEN, the DESRES and the first 2 μs Tinker-HP data set on the first two PCA components of the first 2 μs of the Tinker-HP data set (Fig. 1a and b). One can see that, in this space, the Tinker-HP adaptive scheme already captured the RIKEN and DESRES major main PCA features. It also appears that the RIKEN trajectory sampled a portion of conformational space close to the Tinker-HP data set while the DESRES trajectory seems to explore only the area that is most sampled by Tinker-HP. The same procedure was applied for the PCA components and associated data of the entire Tinker-HP data set (Fig. 1c and d) and it is striking that a much larger portion of conformational space has been sampled by our adaptive scheme. Additionally, we also projected the same data sets on the first two principal components of the RIKEN trajectory which gives the same justification of the larger sampling obtained by our method (see Fig. 4 in the ESI†).

**Fig. 3:**
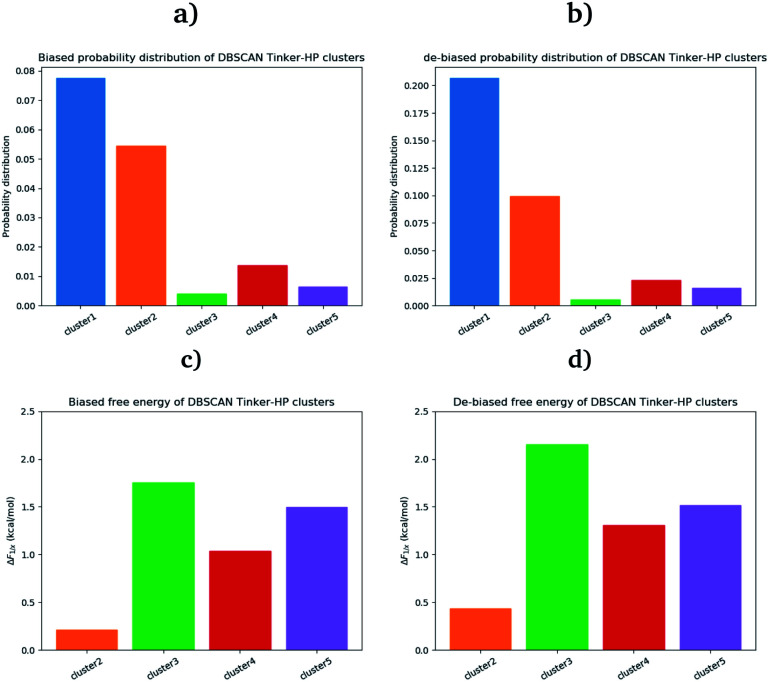
Biased (a) and unbiased (b) probability distribution of DBSCAN Tinker-HP clusters. Biased (c) and unbiased (d) relative free energies of the DBSCAN Tinker-HP 15.14 μs clusters, with respect to cluster 1.

As a preliminary conclusion, we can say that our adaptive sampling strategy allowed us to generate a multi-microsecond polarizable MD simulation that sampled a vast area of the free energy landscape. In addition, we analyzed the Root Mean Square Deviation (RMSD) on protein backbones *versus* the radius of gyration (see Fig. 5 in the ESI†) for the AMOEBA 15.14 μs. It revealed large conformational changes. Variations for the radius of gyration are about 2 Å, while the variation is 1 Å for non-polarizable conventional MD. Such plots are very useful to understand one key question: what makes the AMOEBA results different? Is it the choice of PFF (*vs.* n-PFF) or is it the choice of adaptive sampling strategy. In order to provide a fair (and somewhat quantitative) comparison between the FFs and to de-correlate the effects of the FFs themselves from the gains due to adaptive sampling, we limit ourselves to structures with a reweighting score (see the section above) greater than 1 as it is the score of the frames visited during a conventional MD simulation and as frames with scores lower than 1 are the ones that have been favored by the adaptive algorithm to maximize exploration. 3/4 of the points are therefore removed using this criterion offering a view of the performance of the adaptive sampling. The plot representing the remaining point is presented in Fig. 3 (ESI)† for AMOEBA and it can be directly compared to the RIKEN plot for example. Clearly differences exist between AMBER and AMOEBA results, and they also come from the choice of FF. In addition, important changes are also observed in different important areas of the protease such as the dimerization site. The RMSD of the protein backbone *versus* the RMSD of the chain A dimerization site (see Fig. 6 in the ESI†) depicts large fluctuations between 6 and 7 Å. DESRES and RIKEN trajectories exhibited only 2 Å, which is in the order of the size of the observed PCA features. Overall, these first observations of the differences between the non-polarizable and the polarizable simulations motivate a further analysis of the different simulations.

### 3.4 Unsupervised clustering and extraction of the unbiased relative free energy between representative domains

First, if the PCA analysis reveals useful information, a proper clustering of the produced ensembles is a more precise and quantitative framework to discuss differences between simulations and possible new features captured by the AMOEBA force field. Therefore, we applied to all trajectories the density-based spatial clustering of applications with the noise (DBSCAN) method.^45^ DBSCAN is an unsupervised machine learning algorithm that groups together data in clusters according to their density. It has the particularity to label points as noise if they are not in a dense region and are then not assigned to any cluster. DBSCAN is particularly well suited in our case as it is especially designed to target arbitrary shape clusters. To evaluate the density, DBSCAN uses two parameters, *ε* the distance at which two points are considered to be neighbors and MinPts the minimum number of points needed to define a cluster. *ε* was chosen using the nearest neighbor graph procedure, *i.e.* by plotting the distance to the nearest n-neighbor for each point, ordered from the largest to the smallest value, and evaluating *ε* for which the graph starts forming an elbow. For a given *ε* we then scanned different values of MinPts until relatively large clusters covering a wide range of the space are found. In practice we evaluated the distance to the 4th nearest neighbor on the 4 dimensions composed of the first four 15.14 μs principal components generated by PCA (see Fig. 7 in the ESI†). For DESRES and RIKEN, after being aligned to their respective PDB, the structures were projected on this 4D space.

Our choice of using the AMOEBA 15.14 μs PCA components as the starting point of the clustering is driven by the conformational diversity brought about by the coupling of the PFF and the adaptive sampling scheme. For visualization, clusters are then projected on the first two principal components (Fig. 2). To evaluate the quality of the clustering we used three scoring methods for unknown labeled data:^46^ Silhouette coefficient, Calinski–Harabasz and Davies–Bouldin indices. These indices confirmed our parameter optimization procedure and the high quality of the clustering. Our new adaptive sampling scheme has the main advantage of offering access to true statistical properties such as free energies. To understand the cluster stability, the free energies for each cluster are computed (Fig. 3c and d) through the evaluation of the probability distribution over the total number of structures. Notice that, since not all the structures are part of a cluster, the cluster probabilities do not add up to one. The unbiased probability distribution (Fig. 3a and b) is estimated with the de-biasing procedure explained in the previous section. The de-biasing step preserves the trend between clusters but increases the probabilities. It means that the five clusters were disadvantaged by the adaptive sampling. For example, the biased simulation assessed an 8% probability for the presence of cluster 1, which should have contained, in an unbiased simulation, 20% of the configurations. Besides, cluster 1 is indeed the most explored region by both DESRES and RIKEN. Hence, the algorithm managed to disadvantage this part of the conformational space which is what we could have expected as it favored intermediate transition areas to the detriment of dense regions in order to discover new regions. The effect of the polarizability on structural properties such as volumes and RMSF is further depicted in the next section. Overall, our approach demonstrated our capability to reach high-resolution conformational space exploration using a PFF. We identified 5 different clusters using AMOEBA (see Fig. 2). While some of these states were already identified in previous n-PFF simulations (RIKEN and DESRES), we found two new non-negligible conformations (according to Fig. 3) that can be critical, *e.g.*, for the computation of thermodynamic properties and finally guide further ensemble docking simulations and/or to help to interpret experimental results.

## 4 Correlation with experimental data: structural markers for protomer activity and new features

### 4.1 Markers of the structuring of the oxyanion hole

To ensure the validity of our AMOEBA simulations, we compared our computed properties with available experimental data. Since the beginning of the COVID-19 pandemic various X-ray structures have been released (PDB: 6Y84, 6LU7, 6Y2G, …).^3,47,48^ They provided important insight on specific interactions between residues as well as structural information about the active site. To be consistent with RIKEN simulations we used as reference the same PDB: 6LU7.^3^ Note that DESRES used another PDB, 6Y84,^47^ which we used as a reference in the computation of its properties. Crystal structures have been projected on the first two PCA components of the Tinker-HP simulations (see Fig. 8 in the ESI†).

**Fig. 4:**
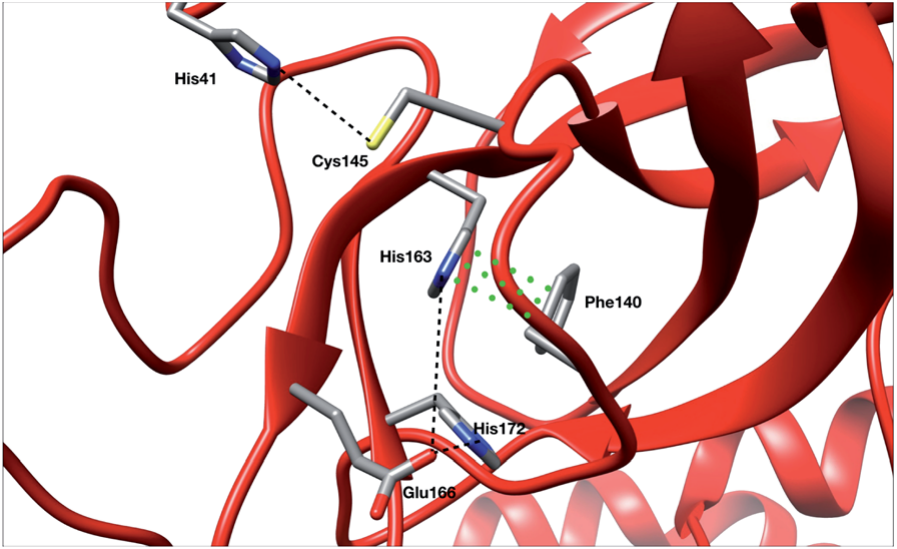
Representation of the π–π stacking interaction between His163 and Phe140 residues (green points) and of several distances of interest which are responsible for the stability of the active site (black dashed lines).

Recently, Zhou *et al.* published an experimental study of the apo structure (PDB 1UJ1)^49^ at physiological pH. They found several features allowing for the characterization of the presence of the oxyanion hole structure which is a key structural element of the activity of each protomer. In particular, they proposed to monitor the distance between Glu166 and His172 and the π–π stacking between Phe140 and His163. The definitions of these structural markers are not new and were initially also discussed for the SARS-CoV-1 M^pro^.^50,51^ The oxyanion hole is responsible for the stabilization of the substrate in the active site and is of crucial importance for the enzyme's kinetics and activity. Indeed, the substrate binding site is composed of 4 pockets labelled S1 to S4 with the S1 pocket involving very conserved residues such as Glu166, His172, His163 and Phe140. The oxyanion hole of the cysteine protease encompasses backbone amides (Gly143, Ser144, and Cys145) while residues 138 to 145 form the so-called oxyanion-binding loop.^48,51,52^ The existence of this latter is responsible in part for the structuring of the S1 pocket.^51^ When the stacking and the Glu166–His172 interaction are broken, a rearrangement occurs leading eventually to the collapse of the oxyanion hole. In this case, Glu166 potentially interacts with His163 instead of His172. In other words, strong interactions of Glu166 with His172 associated with a Phe140–His163 stacking are consistent with a structured oxyanion hole, and can be used as a marker of the activation of the enzyme protomer. Inversely, a strong interaction of Glu166 with His163 would rather be a marker of the protomer inactivation linked with a collapse of the S1 substrate-binding pocket. Of course, such analysis is only interpretative, the oxyanion hole structuring being far more complex. However, it has been shown to be useful since the initial studies on the SARS-CoV-1 main protease.^51^ In practice, the absence of a well-structured oxyanion hole leads to the inhibition of the enzyme's activity. Experimentally, it is known that the M^pro^ monomeric form is inactive while the active form is a homodimer containing two protomers.^53^ In the holo state of SARS-CoV-1, the first protomer is active while the second one is found inactive.^54^ For SARS-CoV-2, a pH = 6 crystal structure (PDB: 1UJ1)^53^ predicted a strong asymmetry of the protomers with an inactive conformation for one of the protomers linked to a broken Glu166 and His172 interaction. However, the inactivity of one of the protomers is still a hypothesis as crystallographic studies of the dimer in the space group *C*2 encounter difficulties in capturing the details of each individual protomer. Indeed, data are only available on one of the protomers in the asymmetric unit which always leads to the more ordered conformation and therefore to the most active one. Concerning the apo state, recent experimental results lead to a potential low activity of the apo dimer linked with an observed destructured oxyanion hole.^49^ It is important to point out that distances/markers exhibit a distribution of different values centered around a maximum of frequency due to the liquid conditions that differ from the crystal ones (Fig. 4).

Then we investigated these markers. To study the Phe140–His163 stacking interaction, we use a stacking-index developed by Branduardi and Parrinello^55^ who described it as a product of 2 Fermi functions, one considering the radial dependence, and the other the angular dependence of the interaction. The model provides an index ranging from 0 for a non-stacked interaction to 0.6 for a perfect one. The Glu166 interactions and π–π stacking were thus calculated for both chains of all RIKEN, DESRES and Tinker-HP structures and then classified into histograms. Finally, each histogram has been unbiased (*i.e.* reweighted) and extrapolated using a univariate kernel density estimator. Final results are given in Fig. 9 of the ESI.† Furthermore a 6 μs adaptive sampling simulation was performed (on the Irene Joliot Curie Machine (TGCC, GENCI, France)) on the monomer species (PDB: 6LU7) and the same features as discussed below (π–π stacking between Phe140 and His163, and Glu166 interactions with both His172 and His163) were calculated. Since the monomer is known to be in an inactive conformation, it helps us to rationalize the behavior observed in our simulations. Results are depicted in Fig. 10 in the ESI.† The preparation and simulation protocols are similar to what we did for the dimer. Therefore, since His172 and His163 are also unprotonated, we minimized the structure up to a RMS of the gradient of 1 kcal mol^−1^ and generated an initial cMD of 200 ns. We then selected 100 random initial structures according to the Adaptive Sampling protocol of structure selection using the PCA, and we performed 6 iterations of 1 μs for a total simulation time of 6 μs.

For the interaction formed by Glu166, in the case of Tinker-HP, we observed an asymmetry between the two protomers. In one protomer the Glu166–His172 interaction is significantly weaker than in the other exhibiting a well-defined marker of a smaller activity of the protomer. This relative non-interaction is in accordance with the results obtained on the monomer which appears to be similar (see ESI Fig. 10†). The situation is more complex in the other protomer where we observe an oscillation between two states, presenting either a formed Glu166–His172 interaction or its absence leading to only some partial activity markers. However, the “interacting” state clearly dominates the statistics. These results demonstrate that the oxyanion hole is only partially organized in the other protomer. This is consistent with experimental data on the apo state^49^ and also with the data on the active protomer of the holo state which shows distances of around 5 Å (see ref. 37 and references therein for a discussion of the different available crystal structures). It is, of course, only one single marker but it could already corroborate the asymmetry observed in the holo state where only one protomer is found to be active,^48^ a similar feature to what was previously observed in SARS-CoV-1.^54^ Based on the analysis of this single marker, we tend to have an inactive first protomer coupled to a second protomer that exhibits some partial but clear activity features (two states) when compared to its inactive counterpart and to the monomer. Similar interpretations can be deduced from the DESRES and RIKEN simulations despite a less clear picture of the His172–Glu166 interactions which appear extremely flexible with more mixed states, especially for AMBER. This is not surprising as Glu–His interactions can be classified as H-bonds, a class of directional weak interactions that are known to be difficult to model using n-PFFs^56,57^ as polarizability contributes significantly to the accuracy of simulations of structures with hydrogen bonds.^15,58^ However, a single distance is not enough to reach a conclusion and should be combined with other markers such as the Glu166–His163 distance. We note here a stronger asymmetry of such distances in protomers for DESRES while in the case of RIKEN and Tinker-HP we could again observe a mixture between interacting/non-interacting states. However, this second marker should be carefully considered as a direct comparison with our monomer simulation (see ESI Fig. 10†) shows that this distance criterion is less well-defined for discussing the protomer “activity” than the Glu166–His172 distance. Since our monomer is known to be inactive, it could be deduced that this marker should always be associated with the evaluation of the Glu166–His172 distance. In practice, one should look at the relative strength of these interactions and the Glu166–His163 distance here appears to be clearly longer than the Glu166–His172 ones. Glu166–His163 distances appear consistent with data on the active protomer of the holo state which shows distances going beyond 6–8 Å (see ref. 37 and references therein for a discussion of the different available crystal structures). In that connection, a better conservation of the catalytic dial is observed in the RIKEN and Tinker-HP simulations with a smaller Cys145–His41 distance compared to DESRES (see ESI Fig. 9†). The active site of the M^pro^ protease comprises a catalytic dyad composed of residues Cys145 and His41. X-ray crystal structures of SARS-CoV-1 (ref. 51 and 52) found a Cys145–His41 distance between 3 and 3.9 Å. In comparison, our simulations revealed distances of around 4 Å while AMBER and DES-AMBER distances are, respectively, around 4.5 and 6–7 Å. Regarding the relatively small differences between the SARS-CoV-1 and SARS-CoV-2 main proteases, AMOEBA results appear closer to experimental data.

Finally, a last marker is studied to confirm our observations: the π–π stacking between Phe140 and His163. Results are depicted in Fig. 9 in the ESI.† Tinker-HP does not capture this stacking in one protomer while again two mixed-states (stacked and un-stacked) are observed in the other protomer. The same observations can be made for DESRES and RIKEN although the states are less well defined in connection with the well-known difficulty of capturing π–π stacking with n-PFFs.^59^ Despite these differences, the 3 simulations appear consistent. Overall, our initial conclusion stands: we describe an asymmetric situation where one protomer is fully inactive and the other shows some partial activity features. It is important to point out that these results are not artificial and linked to our starting structure. Fig. 11 of the ESI† shows the convergence of the stacking marker over the 15.14 μs simulation. If protomer 1 is clearly not evolving over the simulation, protomer 2 evolves slowly towards the discussed 2 state organization. Overall, our results are compatible with the description of the apo crystal structure by Zhou *et al.*^49^ who observed an incomplete structured oxyanion hole exhibiting several mixed states of structuring. This highlights the large flexibility of the enzyme discussed in the experimental literature at room temperature.^38^ Our data also support the possible strong asymmetry between protomers discussed in the holo state.^53^

### 4.2 Evaluation of the volumes of the enzyme cavities

**Fig. 5:**
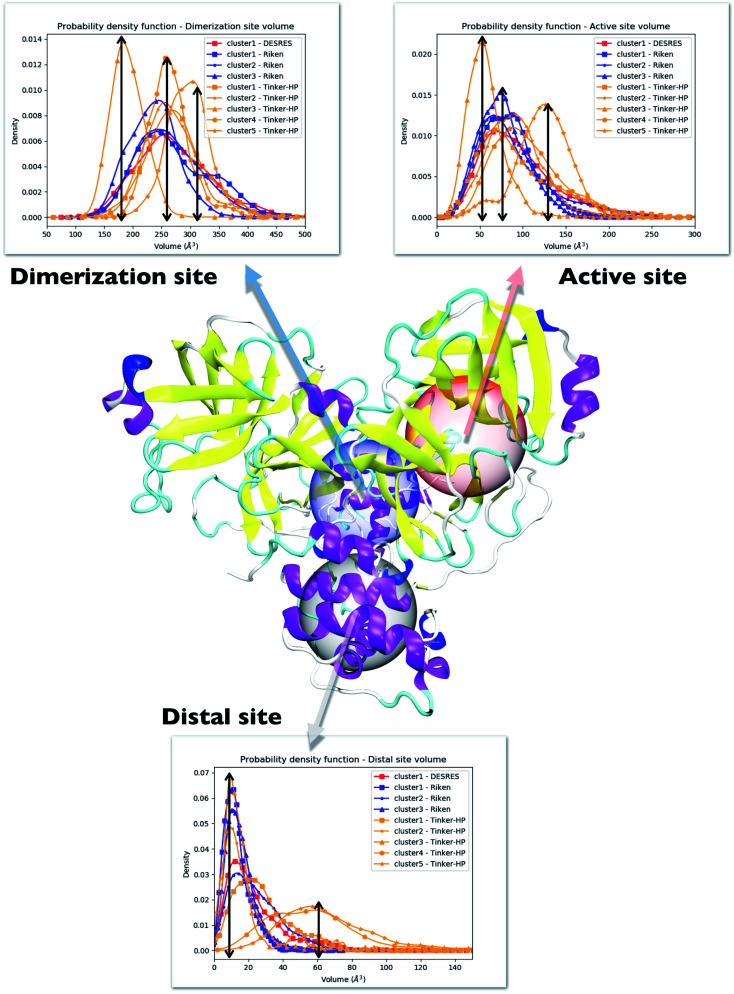
Representation of the 3 cavities considered in this study: the dimerization site, active site and distal site. For each cavity, trends inferred from each cluster are depicted and superposed on three different graphs. Each curve has been unbiased according to the reweighting approach described in this work. Cavity volumes are the sum of volumes found in both protomers. The black arrows link the maxima of frequency to the volume axis to highlight the difference between clusters.

One way to measure some potential global differences between the different simulations is to measure the active site volume in each cluster and to depict the observed trend similarly to the π–π stacking previously. Besides the main active site cavity, the main protease exhibits 2 other cavities: the distal site and the dimerization site. Represented in Fig. 5, these cavities are considered as potential targets for drug inhibition.^60,61^ An accurate description of each of these cavities is essential to the estimation of efficient inhibitors. For each cluster of each dataset, we thus estimated those 3 cavity volumes. Volumes were calculated for each isolated cluster using POVME 3.0 software.^62^ For each cavity, a 1.0 Å grid spacing was chosen. Residues 7–198 and 198–306 and all residues within 3.5 Å from the other protomer were selected for the active, distal and dimerization sites with, respectively, 12 Å, 10 Å and 10 Å. 1000 structures were randomly chosen per cluster for the analysis. When a cluster had less than 1000 structures, we chose all the structures. Detailed information is given in the ESI† on the size of each cluster as well as their relative size (see Table 1 in the ESI†). Similarly to the π–π stacking and the Glu166 distances, we used the univariate kernel density estimator on the volumes. The final volumes are depicted in Fig. 5. Additionally, each cluster has a normal distribution supporting the quality of DBSCAN clusters. Different trends appear, represented by black arrows. For the 3 cavities, we observed a similarity between the single DESRES cluster, clusters 1 and 2 from RIKEN and Tinker-HP's clusters 1 and 2. Agreement is also found with volumes obtained by Sztain *et al.* using a Gaussian accelerated MD (GaMD) enhanced sampling strategy coupled with AMBER ff14SB^8^ which also match these results confirming the importance of simulating long enough in conventional MD. Overall, while Tinker-HP clusters 1 and 2 are in good agreement with RIKEN and DESRES clusters, our clusters 3, 4 and 5 appear to be different and specifically highlight the importance of the PFF choice, *i.e.* these data are not obtained using enhanced sampling coupled with non-PFFs.^8^ As we pointed out earlier, differences indeed occur between clusters and between different datasets, going in the same direction of the previous analysis of the π–π stacking between residues Phe140 and His163 in chains A and B. For Tinker-HP, we observed a contraction for the three cavities in cluster 3 while in cluster 4 and especially cluster 5, we observed a strong difference with a non-negligible increase of the cavity volumes. Cavities from clusters 4/5 depict stronger volume fluctuations when using the AMOEBA PFF. While cavity volumes obtained from AMBER/DES-AMBER simulations and from clusters 1 and 2 from AMOEBA simulations are in agreement, the AMOEBA results clearly capture an additional feature not captured by the DES-AMBER and AMBER simulations. This information could be important for designing potential new inhibitors.

**Table 1:** Average and standard deviation of the number of water molecules around His163 and His41 residues in DES-AMBER, AMBER and AMOEBA force field simulations (pH 7.4)

	His163		His41	
	Protomer 1	Protomer 2	Protomer 1	Protomer 2
DES-AMBER	0.14, *σ* = 0.48	0.77, *σ* = 0.44	4.01, *σ* = 1.17	1.61, *σ* = 0.75
AMBER	0.49, *σ* = 0.57	0.44, *σ* = 0.41	2.38, *σ* = 1.11	2.25, *σ* = 1.23
AMOEBA	0.31, *σ* = 0.51	0.13, *σ* = 0.34	1.48, *σ* = 0.99	1.62, *σ* = 1.06
Experiments^38,49,50,53^	0 or 1		1	

**Fig. 6:**
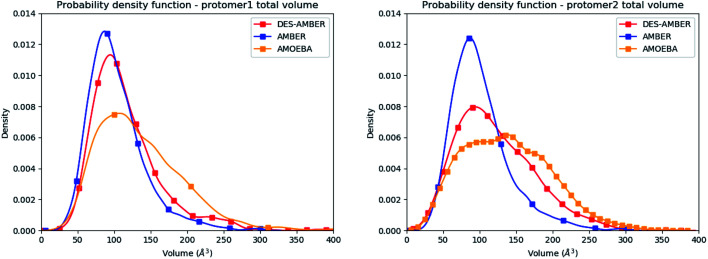
Graphical representation of the distal + active sites for protomer 1 (on the left) and protomer 2 (on the right) for the DESRES, RIKEN and Tinker-HP simulations.

Consequently, since strong differences between methods are observed in the volume evaluations of the different clusters, it is interesting to estimate the global protomer volumes if one wants to try to capture further the discussed asymmetry. Protomer volumes can be found in Fig. 6. Protomer 1 (predicted to be non-active) depicts a strong gaussian behavior while protomer 2 (predicted to be oscillating between an active and a non-active state) is characterized by a spread gaussian with more important associated volume compared to protomer 1. This increase of volume is therefore concomitant with the previous asymmetry related to the various discussed structural markers. It is worth noting that this asymmetry is also found for the DESRES simulation but to a lesser extent compared to that for the AMOEBA Tinker-HP simulations. Concerning the RIKEN dataset, this feature is not found as both protomers depict a similar gaussian trend with very similar values.

### 4.3 Analysis of the local fluctuations: high flexibility of the C-terminal region

**Fig. 7:**
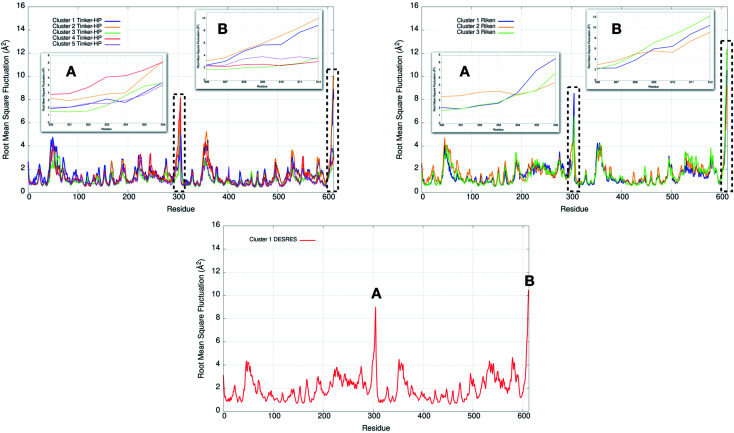
Representation of the RMSF for each cluster of each simulation (Tinker-HP, RIKEN and DESRES). Zoomed-in images of both chains (A and B) are represented in subgraphics and correspond to the C-terminal end where the most important fluctuations are found (residues 300 to 306 for chains A and B).

**Fig. 8:**
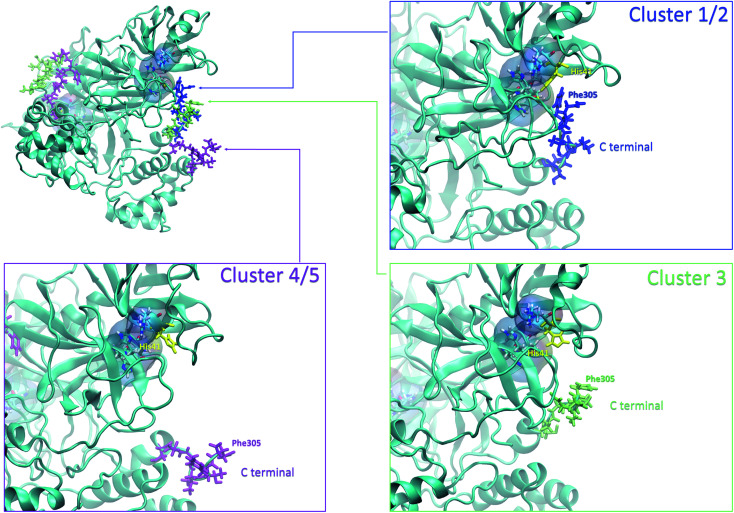
Representation of the 3 possible states of the C terminal end. The whole protein is presented in ice blue. The C-terminal end presented in sapphire blue depicts most of the states in clusters 1 and 2, where the Phe305 residue of the C-terminal region is stacked with His41 of the catalytic site. The C-terminal end presented in lime depicts most of the states in cluster 3, and the one presented in purple depicts most of the states in clusters 4 and 5.

Finally, it is also possible to study local fluctuations in the structural dynamics of the M^pro^ dimer system to uncover other types of difference between datasets. We calculated the fluctuation of residues in each cluster on the same 1000 previously randomly chosen structures per cluster using the Root Mean Square Fluctuation (RMSF). These were calculated on the 5 clusters from Tinker-HP (AMOEBA), the 3 clusters from RIKEN (AMBER) and the single cluster from DESRES (DES-AMBER). Results are depicted in Fig. 7. The most interesting fluctuation as well as the main differences between clusters originates from a different spatial rearrangement of the C-terminal region of the protein (*e.g.* residues 300 to 306 on chains A and B of the dimer). In fact, this region is highly dynamical, which is in accordance with experimental X-ray observations where the electron density of the C-terminal domain was insufficient for backbone tracing, suggesting the flexibility of this region.^49^ Visual enlargements of this region are provided in the sub-graphics of Fig. 7 for chains A and B that do not differ significantly. Cluster 1 from the DESRES simulation depicts the same fluctuation as cluster 1 from the RIKEN simulation. This behaviour of the C-terminal region in these two clusters is characterized by a π–π interaction between Phe305 and His41, eventually blocking the access of any ligand to the active site. When the C terminal region does not interact with His41, it adopts an unfolded configuration which shows the high flexibility of these terminal amino acids. Structural representations can be found in Fig. 8. As this event is observed on the active site of only one chain and not both of them, it could be another marker of the previously mentioned protomer inactivation. We also observed such fluctuations in clusters 1 and 2 extracted from our Tinker-HP/AMOEBA simulations. However, in cluster 1, while the Phe305–His41 π–π interaction is indeed observed, we measure a lower fluctuation of chain A for cluster 1. It corresponds to a weaker interaction between Phe305 and His41 as configurations where the C-terminal branch is less structured are preferred. A similar feature is observed for cluster 2 of RIKEN, but with an inversion of fluctuation peaks between A and B. Overall, clusters 1 and 2 obtained from the Tinker-HP and RIKEN simulations appear relatively similar in the PCA space. They correspond to clusters where the C terminal region can oscillate between two states: one with a π–π stacking interaction between Phe305 and His41, and another with a less structured C-terminal branch with higher flexibility. Clusters 4 and 5 from our Tinker-HP simulations and to a lesser extent RIKEN's cluster 3 correspond to another configuration of the C-terminal region. Representative pictures are provided in Fig. 8 for each cluster C-terminal conformations. In these clusters, the C-terminal region appears more preserved/organized as it is localized further from the active site. To summarize the discussion concerning this specific feature, the high C-terminal flexibility observed in the X-ray experiments can be traced back to a modulated access to the active site linked to the absence of π–π stacking between Phe305 and His41. In other words, the C-terminal region of the fully inactive protomer is shown to oscillate between several states and one of them directly interacts with the other protomer active site. Such interaction tends to block the active site access, therefore modulating down the activity of the potentially most active site. This high flexibility is captured by both RIKEN and Tinker-HP, exemplifying the importance of the local conformational sampling and supporting the experimental analysis of a full inactivation of the apo state.^49^

## 5 Comparative ligandability analysis: searching for cryptic pockets

**Fig. 9:**
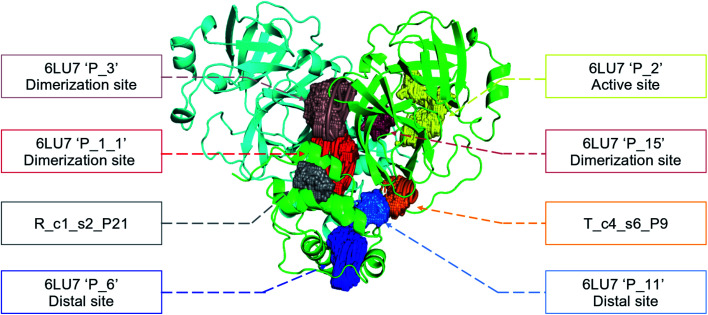
Representation of the pocket locations on the 6LU7 SARS-CoV-2 main protease structure.

**Fig. 10:**
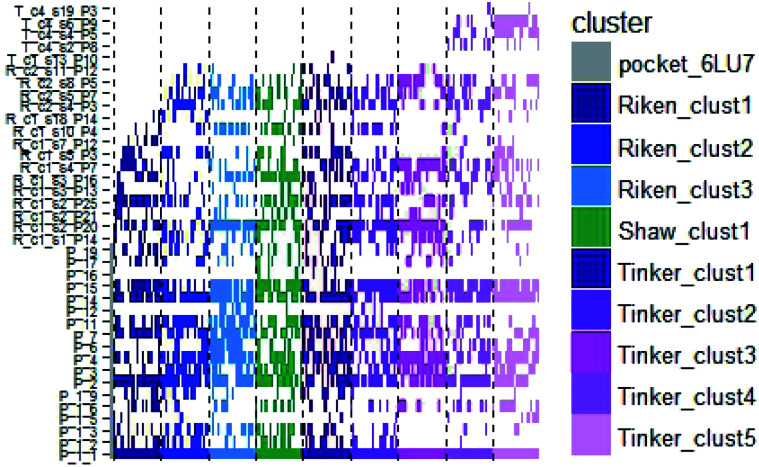
Schematic representation of the detected DoGSite Score pockets within the 6LU7 structure (first column on the left, represented in grey) and 20 structures extracted from each cluster identified within RIKEN (blue gradient), DESRES (green) and Tinker-HP (magenta gradient) simulations.

**Fig. 11:**
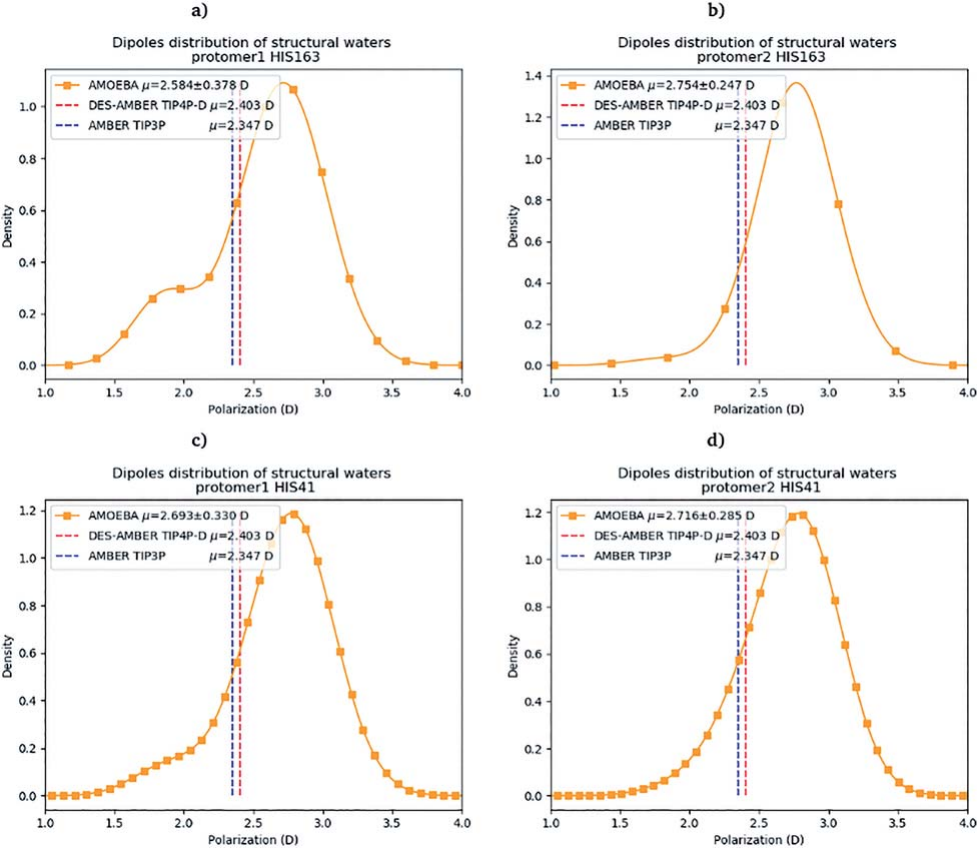
Dipole distribution of water molecules for protomers 1 and 2 around His163 (a and b) and around His41 (c and d).

In order to check if all the previous features could affect the ligandability of the M^pro^ dimer system, we decided to search if new cryptic pockets are detected in each cluster. By taking into account the same sets as for the cavity volume analysis, cryptic pockets were searched using DoGSite Scorer software,^63^ an automated tool for pocket detection and pocket descriptor calculation. DoGSite Scorer detected 18 pockets located on chain A or at the interface of chains A and B of the SARS-CoV-2 protease 6LU7 crystal structure. Among these pockets, 6 are already described in the literature:^8,64^ pockets ‘P_1_1’, ‘P_3’ and ‘P_15’ corresponding to the dimerization site; the ‘P_2’ pocket corresponding to the active site and the ‘P_6’ and ‘P_11’ pockets located in the distal region. These 18 pockets were used as a reference and all pockets detected on the DESRES, RIKEN and Tinker-HP selected structures were assigned to these reference pockets by comparing the list of residues of the different pockets and selecting the reference pocket with the maximum number of common residues. When the maximum number of common residues was lower than 5, and the ratio between the maximum number of common residues and the number of residues in the predicted pocket was below 0.25, the pocket was not assigned to any reference pocket and was defined as a new cryptic pocket. New cryptic pockets were named after the first structure in which they were detected and added to the set of reference pockets. For example, the ‘R_c1_s1_P14’ mentioned in Fig. 11 is the pocket P_14 detected by DoGSite Scorer in structure 1 (s1) of cluster 1 (c1) of the RIKEN (R) simulations. The results of pocket assignation and new cryptic pocket identification are presented in Fig. 10. We observed that the reference pockets previously highlighted as ‘active site’, ‘dimerization site’ and ‘distal site’, except ‘P_6’, are particularly conserved and detected in a large majority of analyzed structures. However, a consequent number of other pockets were also detected: (1) in a few structures such as ‘R_c1_s2_P21’, ‘R_c1_s18_P14’ or ‘T_c4_s19_P3’ or (2) in many structures, such as ‘R_c1_s2_P20’, ‘R_c1_s2_P25’ or ‘R_c1_s4_P7’. Interestingly, only 3 pockets were retrieved in clusters 4 and 5 of the Tinker-HP simulations: ‘T_c4_s2_P8’, ‘T_c4_s5_P5’ and ‘T_c4_s6_P9’. The last one, ‘T_c4_s6_P9’ is of particular interest since its volume is equal to 199 Å^3^ and its druggability score, DrugScore,^65^ reaches 0.62. We repeated the pocket detection and analysis procedure on 100 randomly selected structures (20 for each of the 5 clusters) identified within the Tinker-HP simulations (see Fig. 10 in the ESI†). We observed that the 3 previously identified pockets ‘T_c4_s2_P8’, ‘T_c4_s5_P5’ and ‘T_c4_s6_P9’ were also detected on the structures randomly selected in clusters 4 and 5 of the Tinker HP simulations but also partially in cluster 3. We then evaluated if all the pockets assigned to the ‘T_c4_s6_P9’ pocket displayed similar properties. We observed that the mean volume of these pockets was 215 Å^3^ but few structures presented extreme values far superior to this mean volume (Fig. 12 in the ESI†). Similarly, the DrugScore mean value was 0.37 but with large variations among the structures and the clusters (see Fig. 13 in the ESI†). For comparison, we also computed the DrugScore value distribution for each newly identified pocket, *i.e.* pockets that were not detected in the 6LU7 structure (Fig. 14 in the ESI†). One pocket, ‘R_c1_s2_P21’, displays peculiar properties with a mean druggability value of 0.6 and a mean volume value of 150 Å^3^ which seems to indicate that this pocket may only accommodate very small compounds. The discovery of the ‘T_c4_s6_P9’ pocket is thus a very promising result, but one that underlines the necessity of carefully selecting one or several structure(s) in which the pocket properties are optimal for further *in silico* investigations to identify small molecules able to modulate the SARS-CoV-2 protease activity. All the pockets discussed herein are represented within the 6LU7 structure in Fig. 9.

## 6 Solvation analysis: the importance of including explicit polarization effects in water

Water molecules play critical roles in enzyme and protein functioning. In fact water can be a product or a reactant in condensation and hydrolysis reactions, a transition state intermediate in chemical reactions and a structural element at the molecular level. In the lattermost case, water interconnects the protein through hydrogen bonds in order to maintain and stabilize the positions of the residues and the fold.^66^ Previous experimental studies on SARS-CoV-1 and SARS-CoV-2 have shown that one structural water molecule was conserved within the main protease of the two viruses and interacts with the cyclic nitrogen of His41.^38,51,52^ A recent crystallographic study on SARS-CoV-2 suggests that another water molecule could be observed around His163.^49^ In order to calculate the number of water molecules inside the active site and in proximity of His41 and His163 of both protomers, we have created a virtual sphere of 4 Å, centered on the nitrogen of each of the two concerned histidines and have calculated the number of water molecules inside the active site of each protomer over time. Fig. 11 shows the dipole distribution of structural water molecules for protomers 1 and 2 of His163 (a and b) and His41 (c and d). The AMOEBA results are striking. They show that (i) the water molecules in each of the two protomers' active sites are highly polarized, and (ii) the AMOEBA distribution of the water molecules is significantly different from the ones observed in the DESRES TIP4-D (DES-AMBER) and RIKEN TIP3P (AMBER) trajectories. High polarization has been shown in past studies to be a common feature of structural water molecules that exhibit high dipole moments.^67^ In practice, the average dipole moment having the highest density with the AMOEBA force field is located around 2.9 D while for the DES-AMBER and AMBER n-PFFs, the water dipoles are fixed at 2.403 D and 2.347 D, respectively (see Fig. 5). Since AMOEBA dipole moments are not fixed, we observe strong polarization fluctuations due to water traffic inside the catalytic region. Fig. 15 in the ESI† presents the number of structural water molecules for protomers 1 and 2 of His163 (a and b) and His41 (c and d). All trajectories show a highest density for no water molecules within a distance of 4 Å from protomer 1 of His163. However, this observation is different for protomer 1 of His41 where Tinker-HP trajectories found a highest density for the presence of one water molecule while it was 2 molecules for RIKEN's and 4 molecules for DESRES's trajectories. A non-symmetric distribution of water molecules compared to protomer 1 is found for protomer 2. Tinker-HP and RIKEN trajectories do not predict the frequent presence of water molecules within the chosen distance from His163, while DESRES's trajectories exhibit a higher density for 1 molecule. Concerning His41 of protomer 2, Tinker-HP's and DESRES's trajectories show a most frequent density of one water molecule, while RIKEN's highest density goes to 2 water molecules, and slightly less for 1 molecule. These observations demonstrate that water polarization intensively fluctuates inside the confined active site, suggesting a dynamic role of polarization on water traffic that strongly influences water molecule interactions with His163 and His41 of each of the two protomers. However these interactions are not distributed symmetrically between protomers. So is it compatible with experimental data? Again, relatively detailed X-ray data exist for other coronaviruses including SARS-CoV-1 where the role of histidines has been extensively discussed.^51,52^ The presence of a structural water molecule around His41 is always confirmed. For SARS-CoV-2, papers describing the M^pro^ protease structure in its apo state^38,49^ under physiological pH conditions also discuss the presence of such molecule found near the catalytic dyad (His41). However, the interaction of the structural water molecule with His163 appears to only be proposed in Zhou *et al.*'s report.^49^

Concerning the precise predicted water count around His41, AMBER and DES-AMBER have on average a higher number of structural water molecules (2.38 to 4.01 at the most) compared to AMOEBA which predicts the presence of 1.5 water molecules, more in line with accumulated experimental data. Fig. 15 in the ESI† shows that the non-polarizable simulations capture frequent configurations with up to 4 water molecules which could be a consequence of the non-inclusion of the polarization effect leading to a weaker and constant dipole moment of the water molecules that could generate more water traffic. Compared to His41, all AMOEBA, AMBER, and DES-AMBER analyses found significantly fewer water molecules around His163. In practice AMOEBA found the lowest water count of all methods with an average of 0.13–0.31 molecules around His163, while the higher trends observed for His41 are still present for all n-PFFs except for one protomer of DES-AMBER that exhibits 0.77 molecules (see Table 2). Clearly, the presence of a structural water molecule around His163 seems less probable for all simulations (under the present pH conditions) and in competition with the water traffic entering the measurement sphere. The dipole distribution of water molecules offers further analysis as it is found to be slightly larger for His163 and associated with a smaller density of highly polarized total dipole moments confirming the trends. In any case, the presence of water in the active site thus appears consistent with the need for a water molecule to model the enzyme reaction mechanism.^38,68^

**Table 2:** Average and standard deviation of the number of water molecules around His163 and His41 residues using AMOEBA for simulations at pH 7.4 and 6

	His163		His41	
	Protomer 1	Protomer 2	Protomer 1	Protomer 2
AMOEBA pH 6	0.37, *σ* = 0.65	0.27, *σ* = 0.57	1.95, *σ* = 1.04	1.42, *σ* = 0.97
AMOEBA pH 7.4	0.31, *σ* = 0.51	0.13, *σ* = 0.34	1.48, *σ* = 0.99	1.62, *σ* = 1.06
Experiments	0 or 1		1	

## 7 Further simulation at lower pH: impact of His172 protonation

From the past studies on SARS-CoV-1 (see ref. 51 and references therein) we know that the activity of the main protease system is pH dependent. While its activity is lower at low pH and high pH, it is higher at pH close to the physiological human pH (*i.e.* 7.4). Studies performed on the M^pro^ of SARS-CoV-1 show a bell-shaped pH–activity curve^51^ for the enzyme. All proposed simulations (*i.e.* ours and the one from DESRES and RIKEN) were performed using neutral histidine residues. Indeed, one key element of the impact of lowering the pH is the protonation of His172 and His163.^51^ Initially, based on SARS-CoV-1 knowledge, it was thought that if His172 and His163 were not protonated at pH = 8, His172 would be in a protonated state in both protomers at physiological pH (pH = 7.4) since its p*K*_a_ was found to be close to 7.6.^69^ However, differences exist with the SARS-CoV-2 M^pro^, and Verma *et al.* recently showed^37^ that the p*K*_a_ of His172 would be actually lower than anticipated, being about 6.6. Such prediction appears consistent with recent experimental results.^38^ Our proposed simulation setup using neutral histidines is therefore likely to be consistent with physiological pH conditions. In that connection, Verma *et al.* described the critical role of the protonation of His172 on the holo state that would happen at pH = 6 and they showed that it would lead to a partial collapse of the S1 pocket, linked with a strong destructuring of the oxyanion hole.^37^ Thus, it appears critical to investigate the influence of pH on our apo results by performing an additional simulation compatible with pH = 6 conditions. So, in order to propose a starting point for this second simulation, we followed a protocol found in the literature for SARS-CoV-1.^51^ We then selected 15 new structures from our pH = 7.4 simulation (3 structures per cluster). For each structure we then protonated the His172 on both protomers, which initiates the structural transformation from pH = 7.4 to pH = 6. The same simulation protocol (see Section 3.2) was followed and a total of 17 μs of simulation was thus generated using the Jean Zay Supercomputer (IDRIS, GENCI, France). In practice, with enough sampling, the structures should be able to relax. Of course, as pointed out by Verma *et al.*,^37^ other residues could be impacted by lowering the pH but such simulation has strong interpretative interest. We therefore looked again at all the structural markers described for the previous simulation. We first studied the convergence of some of the properties. Fig. 11 in the ESI† shows that the simulation tends to converge more slowly than at physiological pH and starts to do so beyond 14 μs. Clearly, comparisons of both pH situations would not have been possible using nanosecond simulations even if initial local relaxation of the histidine residues appears to have happened at this timescale. Of course, we cannot state that the simulation is fully converged. However, we stopped the computation when the observed structural changes strongly diminished over time within the ensemble, leaving us with enough confidence in the computed properties. The key result obtained from this second long simulation is the strong variation of the activation features present in the previously described inactive protomer. Indeed, while a significant asymmetry between protomers was found at pH = 6 with protomer 1 exhibiting a poor structure oxyanion hole, the situation evolves with the protonation of His172. Indeed protomer 1 now exhibits a mix of several states with different structural markers (see Fig. 16, ESI†). Compared to pH = 7.4, the interaction of His172/163 with Glu166 changed from a H-bond type interaction (neutral His172/163 at pH = 7.4) to a salt-bridge (positively charged His172 at pH = 6).^70^ The stacking index shows that the stacking interaction appears to be weaker than at physiological pH and therefore easier to break and to form (see ESI Fig. 17†). As a result of the protonation, protomer 1 now shows two relatively short maxima for the Glu166–His172 distance (see ESI Fig. 16†) associated with a continuum of values of distances going beyond 6 Å. The protomer 1 Glu166–His172 distance appears to explore a variety of situations including a favorable stacking second minimum which is a sign of a more structured state. However, while some ordered states are found, the absence of stacking is statistically dominant and associated with a striking set of Glu166–His163 interactions. Clearly some really short hydrogen-bonds are found between these residues, a sign of a strong destructuring of the oxyanion hole. These results are in line with the findings of Verma *et al.*^37^ that associated the protonation of His172 with the collapse of the oxyanion loop toward the S1 pocket. However, for the other protomer, our apo results differ a bit from Verma *et al.*'s holo data. Indeed, the situation appears more contrasted. Despite a net destructuring effect, protomer 2 tends also to exhibit a mix of states after protonation. The protomer encompasses longer Glu166–His172 interactions than previously noted at physiological pH and the noticeable appearance of some states with short Glu166–His163 distances is observed. However, in the case of protomer 2, the stacking still statistically partially holds despite the existence of a second peak describing a non-negligible absence of stacking in some configurations. Overall, our computations show that the protomers tend to be both affected by the destructuring effect of the His172 protonation, leading to a more symmetrical situation between destructured protomers. Protonation of His172 definitively increases the dynamical aspect of the protease structure and favors the exploration of different states of the activation markers highlighting the instability of the oxyanion hole leading to the partial collapse of the S1 pocket. The impact of the increased flexibility can be further examined through the comparative RMSF of the two simulated pH states where the mobility of the C-terminal end appears further enhanced (see ESI Fig. 17†). This clearly correlates with our initial remark concerning the sampling, that such lower pH structure is far more complex to simulate than the situation at physiological pH as several states resonate due to the low structuring of the oxyanion loop. Finally, Table 2 shows the evolution of the solvation around His163 and His41. The number of water molecules found in the AMOEBA simulation tends to increase on both histidine sites compared to pH = 7.4 with more configurations including one and two water molecules for His163 and His141, respectively. If the presence of a structural water molecule is confirmed around His41, a similar presence around His163 tends to be statistically reinforced under these protonation conditions. Clearly these findings have potentially an important impact in drug discovery as the presence of structural water molecules around His141 and potentially His163 would make rational drug design more difficult since the substrate or inhibitors would suffer from steric hindrance.^49^ The use of PFFs could be critical in the evaluation of the free energies of binding of possible drug candidates. Indeed, our data confirm the high plasticity of the active site observed in X-ray structures^38^ at room temperature. Modeling such plasticity including the structuring of the S1 pocket clearly requires the simultaneous capability to accurately evaluate various types of weak interaction including hydrogen bonds, salt bridges and π–π stacking while high-resolution modeling of solvation appears to also be mandatory. Of course, we also showed that extensive sampling beyond the μs-timescale was crucial to deal with such difficult flexible systems.

## 8 Conclusion and perspectives

In this work, designed in response to the urgent need for COVID-19 research, we demonstrated that it is now possible to perform long μs-timescale MD simulations of large biosystems using polarizable force fields such as AMOEBA that are able to account for physical many-body effects. Due to the inherent complexity of the SARS-CoV-2 proteins, performing such higher-resolution simulations is important as they could provide additional information about the structural dynamics of virus constituents to the COVID-19 experimental and computational research communities. To do so, we proposed a fully unsupervised adaptive sampling strategy that can be used on any type of computational resources. This automated framework allows for production simulations that benefit from advances in supercomputing and from our recent Tinker-HP HPC massively parallel software enhancements, that can now efficiently handle GPU-accelerated large petascale computers using lower precision arithmetic and MPI. In order to extract new information from this type of simulation, we also provided the necessary steps to remove the bias from (re-weight) the obtained data to collect useful and accurate structural dynamics features. More than 38 μs of all-atom MD simulation of the M^pro^ enzyme in its apo (ligand-free) state was produced using the AMOEBA polarizable force field.

Results were then compared to available state-of-the-art large scale simulation data. The results from the new generation PFF were shown to capture most of the structural dynamics features discussed in the experimental literature, confirming that M^pro^ is probably in a poorly active conformation in its apo state under physiological pH conditions. However, simulations detected some partial activity features in one of the protomers linked to a more structured oxyanion hole. This is consistent with the protomeric asymmetric activity observed in the holo state where only one protomer is found to be active,^48^ a similar feature that was also observed in SARS-CoV-1.^54^ This asymmetry can be related to several structural markers as well as to the total protomer volumes. The active site is found to be highly flexible at room temperature in agreement with recent experimental findings.^38^ Overall, the apo state of M^pro^ clearly appears less organized than the holo state in agreement with experimental results discussed by Zhou *et al.*^49^ A second simulation, including the protonation of the His172 residue to simulate the system under pH = 6 conditions, was performed and tends to confirm the role of the protonation in the collapse of the S1 pocket at lower pH. Under these conditions, the protomeric AMOEBA asymmetry remains although the protomers tend to be notably destructured. The AMOEBA simulations also captured the C-terminal high flexibility feature discussed in the literature.^49^ Flexibility increases at lower pH and tends to further modulate down the activity of the apo state linked with the collapse of the S1 pocket. Striking differences were observed concerning the solvation patterns around the key His41 and His163 residues between AMOEBA and n-PFFs. Overall, the smaller AMOEBA water count around histidines is more in line with experimental data. If the presence of a structural water molecule around His41 is probable at all pH, the existence of a water molecule around His163 tends to be more statistically possible at pH = 6. These results can be explained by the capability of AMOEBA structural water molecules to exhibit an average dipole moment higher than that of bulk water and to explore a wider range of dipoles compared to n-PFFs. Structural water molecules around histidines will clearly affect rational drug design. The use of polarizable force fields could be critical in the evaluation of the free energies of binding of possible drug candidates competing with water to interact with the enzyme. In practice, the M^pro^ enzyme tends to be difficult for molecular mechanics approaches. Indeed, it encompasses all sorts of weak interactions. Therefore, it is not surprising that all the experimentally described features found within the AMOEBA simulations were not necessarily found with the non-polarizable simulations. Such systems tend to require both an accurate force field and an extensive sampling strategy as it is obvious that a few ns of PFF MD alone would not provide insights into a system where the statistical convergence is challenging due to its plasticity. These results provide a first direct validation of the stability of the AMOEBA polarizable force field and clearly demonstrate its applicability at long timescales. Besides correlating with experimental data, our results also show that our adaptive sampling approach coupled with AMOEBA led to enhanced volumes for the active site and to additional potential cryptic pockets as well. As the apo (ligand-free) state has been shown to be a relevant structure at room temperature to perform docking studies,^38^ the new information provided could be useful for drug design. Our simulation data are fully available to the general public. They can therefore be used for further structural analysis and/or as an additional basis for ensemble docking studies.^71^ Indeed, concentrating the GPU computing power on an apo state is useful to “mine” the conformations to obtain an accurate and more statistically converged set of MD binding site conformations that could be selected by a ligand. The new structural information provided here could help to design new drugs or to repurpose existing ones. These data could also be important to understand chemical reactivity at an atomic level *via* hybrid QM/MM simulations.^68,72^ Finally, thanks to the presented divide and conquer strategy, our AMOEBA adaptive MD simulations were shown to be simultaneously computationally competitive and in line with the available experimental data. Using 100 GPU cards, we show that an acceptable and competitive time to solution could be achieved as our “microsecond” results were obtained in a few days on an academic (and multipurpose) supercomputer. It is worth noting that each simulation could have run on full nodes or using more efficient A100 cards. In practice, a similar exploration of the available community data was already achieved in only 2.5 days (Fig. 1). It is also important to note that Tinker-HP can also produce an order of magnitude faster simulation using n-PFFs using GPUs. Since n-PFF simulations are also of great interest, capturing many experimental aspects, our dual-level (n-PFF + PFF) strategy is confirmed. Indeed, an optimal setup consists in first producing a long adaptive non-polarizable simulation that can be further refined with polarizable potentials within additional adaptive iterations. That way, our approach could also use Folding@home COVID-19 community results^73^ as an input (or any available data shared on the BioExcel/Molssi repository) in order to deliver a maximum of potentially new/useful information into COVID-19 research. Indeed, it is important to recall the importance of proposing accurate (and as much as possible converged) simulations of the COVID-19 targets. As a final perspective, we can mention that the present strategy is platform independent and not limited to supercomputers. Therefore, it can also be used at a smaller scale on “cheaper” laboratory GPU clusters which can benefit from the computational power of low arithmetic to obtain local supercomputing capabilities. On the other side of the spectrum, with the coming of the exascale era and the HPC–Artificial Intelligence (AI) convergence, the “big iron” supercomputer systems, and their cloud-computing counterparts, will considerably extend the high accuracy conformational mining capabilities leading to extended possibilities for the *in silico* modeling of complex biological systems.

## Author contributions

T. J. I., F. C., D. El A., and N. L. performed simulations; O. A., T. J. I., and L.-H. J. contributed new code. P. M., T. J. I., J.-P. P, P. R., and L. L. contributed new methodology. N. L., M. M., L. L., F. C., and P. M. contributed analytical tools. F. C., T. J. I., D. El A., N. L., M. M., P. R., and J.-P. P. analyzed data. J.-P. P., P. M., L. L., N. L., T. J. I., F. C. and P. R. wrote the paper; J.-P. P. designed the research.

## Conflicts of interest

There are no conflicts to declare.

## Supplementary Material

SC-012-D1SC00145K-s001

SC-012-D1SC00145K-s002
