# Supplementary figures and images for: High-resolution mining of the SARS-CoV-2 main protease conformational space: supercomputer-driven unsupervised adaptive sampling
Source: Chem Sci. 2021 Feb 2;12(13):4889–907. doi: 10.1039/d1sc00145k (PMC8179654; doi:10.1039/d1sc00145k)

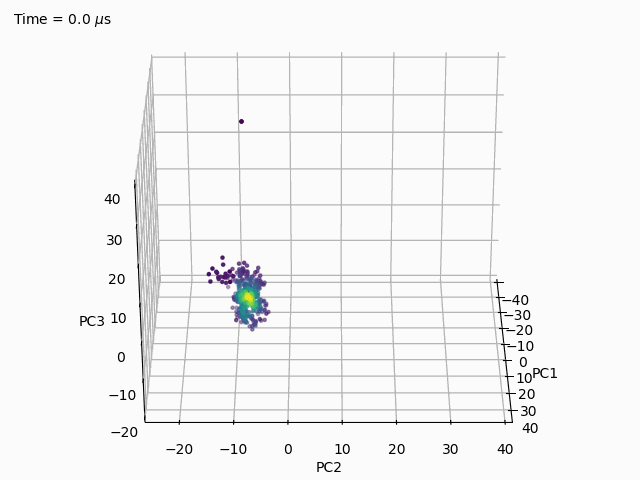

Supplement: SC-012-D1SC00145K-s001 [file SC-012-D1SC00145K-s001.gif]
