# Supplementary material for: High-resolution mining of the SARS-CoV-2 main protease conformational space: supercomputer-driven unsupervised adaptive sampling
Source: Chem Sci. 2021 Feb 2;12(13):4889–907. doi: 10.1039/d1sc00145k (PMC8179654; doi:10.1039/d1sc00145k)
Supplement: SC-012-D1SC00145K-s002 [file SC-012-D1SC00145K-s002.pdf]

Electronic Supplementary Material (ESI) for Chemical Science.
This journal is © The Royal Society of Chemistry 2021

High-Resolution Mining of SARS–CoV–2 Main Protease Conformational Space: Supercomputer-Driven Unsupervised Adaptive Sampling

Théo Jaffrelot Inizan [1,+], Frédéric Célerse[1,2,è +], Olivier Adjoua[1], Dina El Ahdab[1,3], Luc-Henri Jolly[4], Chengwen Liu[5], Pengyu Ren[5], Matthieu Montes[6], Nathalie Lagarde[6], Louis Lagardère[1,4,*], Pierre Monmarché[1,7,*], and Jean-Philip Piquemal[1,6,8,*]

[1]Sorbonne Université, Laboratoire de Chimie Théorique, UMR 7616 CNRS, 75005, Paris, France
[2]Sorbonne Université, Institut Parisien de Chimie Moléculaire, UMR 8232 CNRS, Paris, France
[3]UR EGP, Centre d'Analyses et de Recherche, Faculté des Sciences, Université Saint-Joseph de Beyrouth, Beirut, Lebanon
[4]Sorbonne Université, Institut Parisien de Chimie Physique et Théorique, FR 2622 CNRS, 75005, Paris, France
[5]UT Austin, Department of Biomedical Engineering, TX 78712, USA.
[6]Laboratoire GBCM, EA 7528, CNAM, Hésam Université, 75003, Paris, France
[7]Sorbonne Université, Laboratoire Jacques Louis Lions, UMR 7598 75005, Paris, France
[8]Institut Universitaire de France, 75005, Paris, France.
[*]Contacts : louis.lagardere@sorbonne-universite.fr, pierre.monmarche@sorbonne-universite.fr, jean-philip.piquemal@sorbonne-universite.fr
[+]These authors contributed equally to this work

Supplementary informations

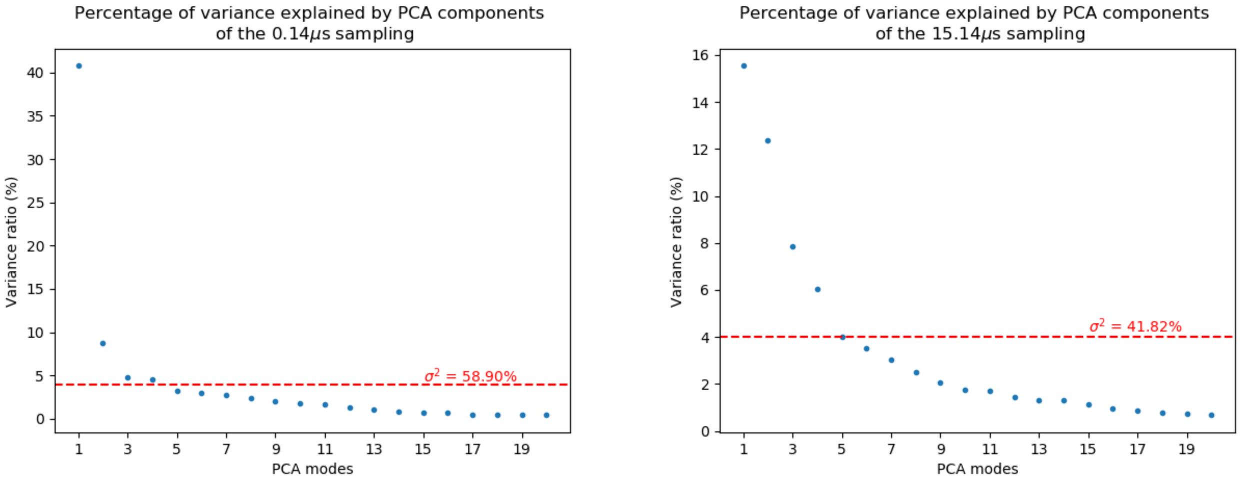

Figure 1. Percentage of variance explained by the first twenty PCA components of respectively 0.14μs **a)** and 15.14μs **b)**. The red horizontal line corresponds to the limit of components with variance ratio more than 4% and their corresponding total variance sum.

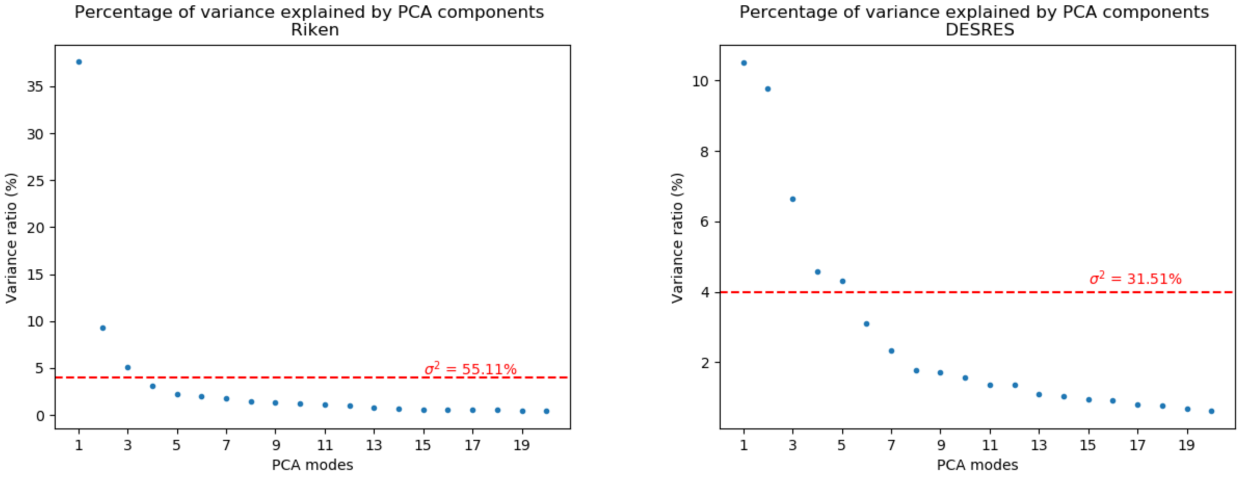

Figure 2. Percentage of variance explained by the first twenty PCA components of respectively DESRES **a)** and Riken **b)**.

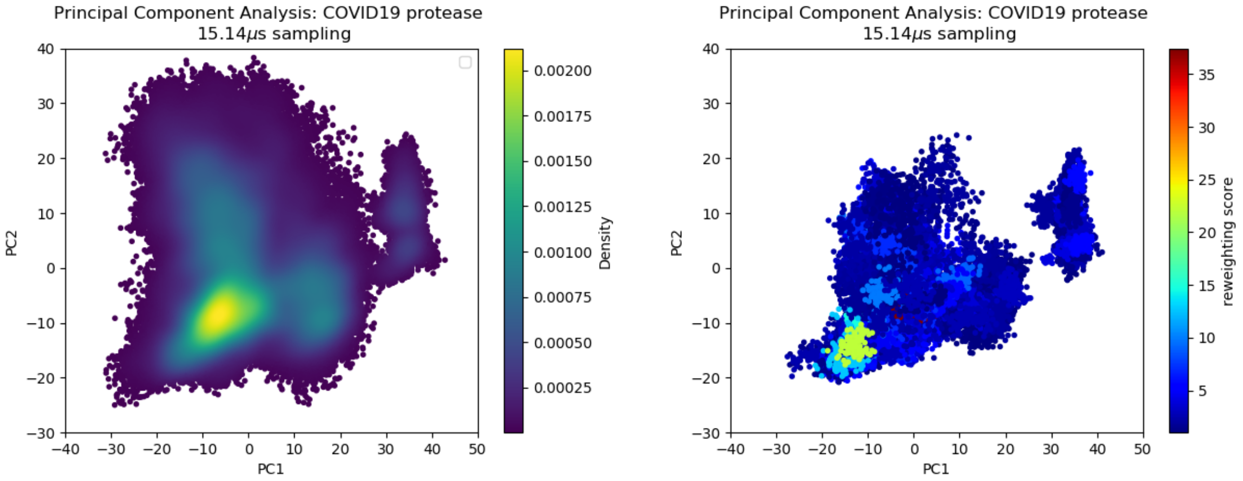

Figure 3. Projection of AMOEBA 15.14μs simulation and colored according to their reweighting score. Only the structure with a score larger than 1 are shown (correspond to a reduced dataset of 33100 structures).

We noticed that the de-biasing scores can be used as an exploratory map of the free energy landscape and adaptive sampling algorithm. Indeed, low scores correspond to atypical configurations that would have not been observed by classical MD (or at least to configurations which, starting from the initial configurations, have been reached only after a crossing of some atypical areas). Figure 3 reveals large areas of such low score configurations, which shows that the adaptive selection has had a strong effect on the exploration of rare events.

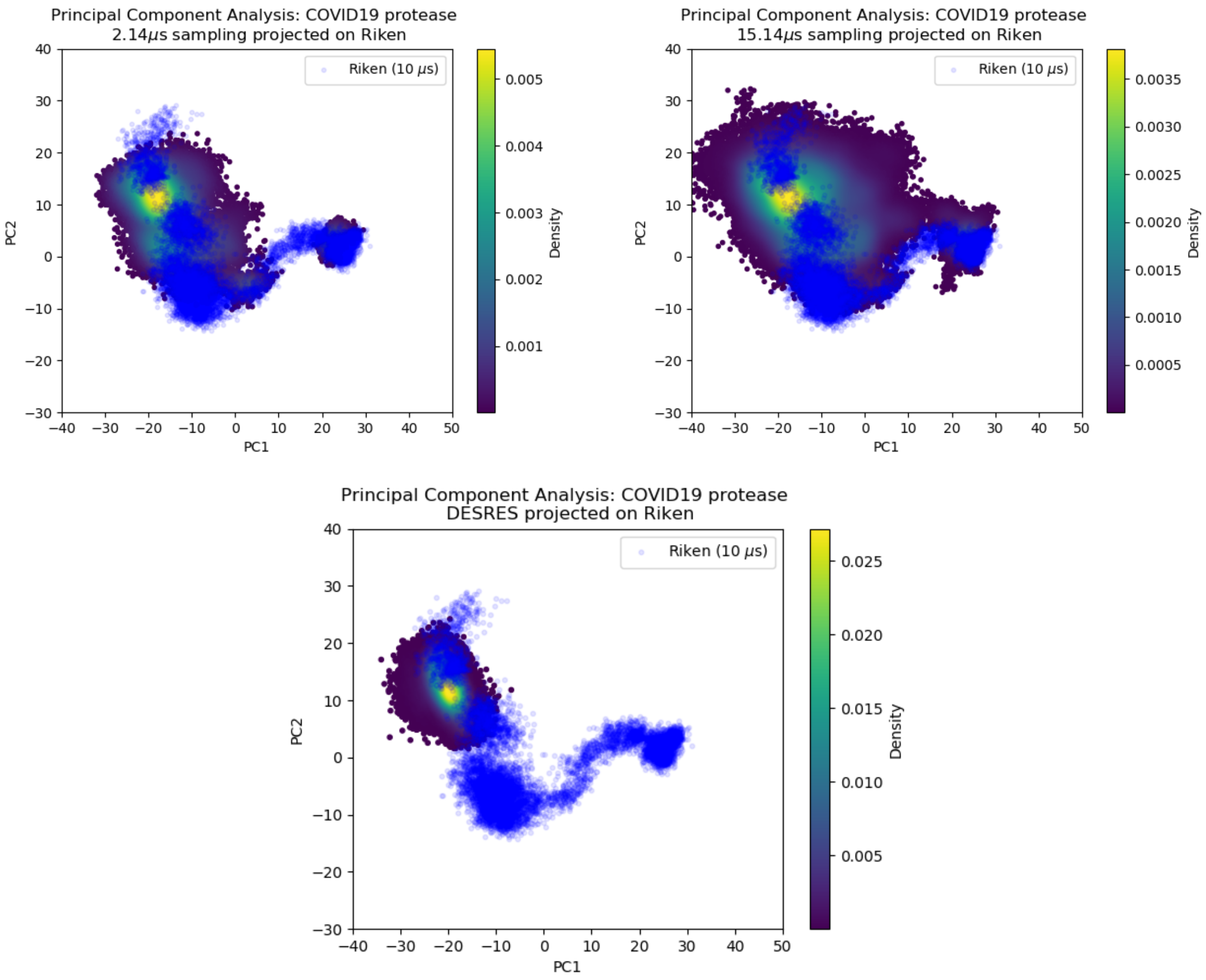

Figure 4. Projection of AMOEBA 2.14μs **a)**, 15.14μs **b)** and DESRES 100μs **c)** on the first two principal Riken PCA components.

	Cluster's size	Reduced cluster's size
cluster1 DESRES	11 731	1 000
cluster1 RIKEN	347	299
cluster2 RIKEN	3 018	1 000
cluster3 RIKEN	2 463	1 000
cluster1 Tinker-HP	11 735	1 000
cluster2 Tinker-HP	8 256	1 000
cluster3 Tinker-HP	622	599
cluster4 Tinker-HP	2 062	1 000
cluster5 Tinker-HP	958	899

Table 1. Full and reduced clusters size used in the cavity volumes, C terminal flexibility and cryptic pockets studies.

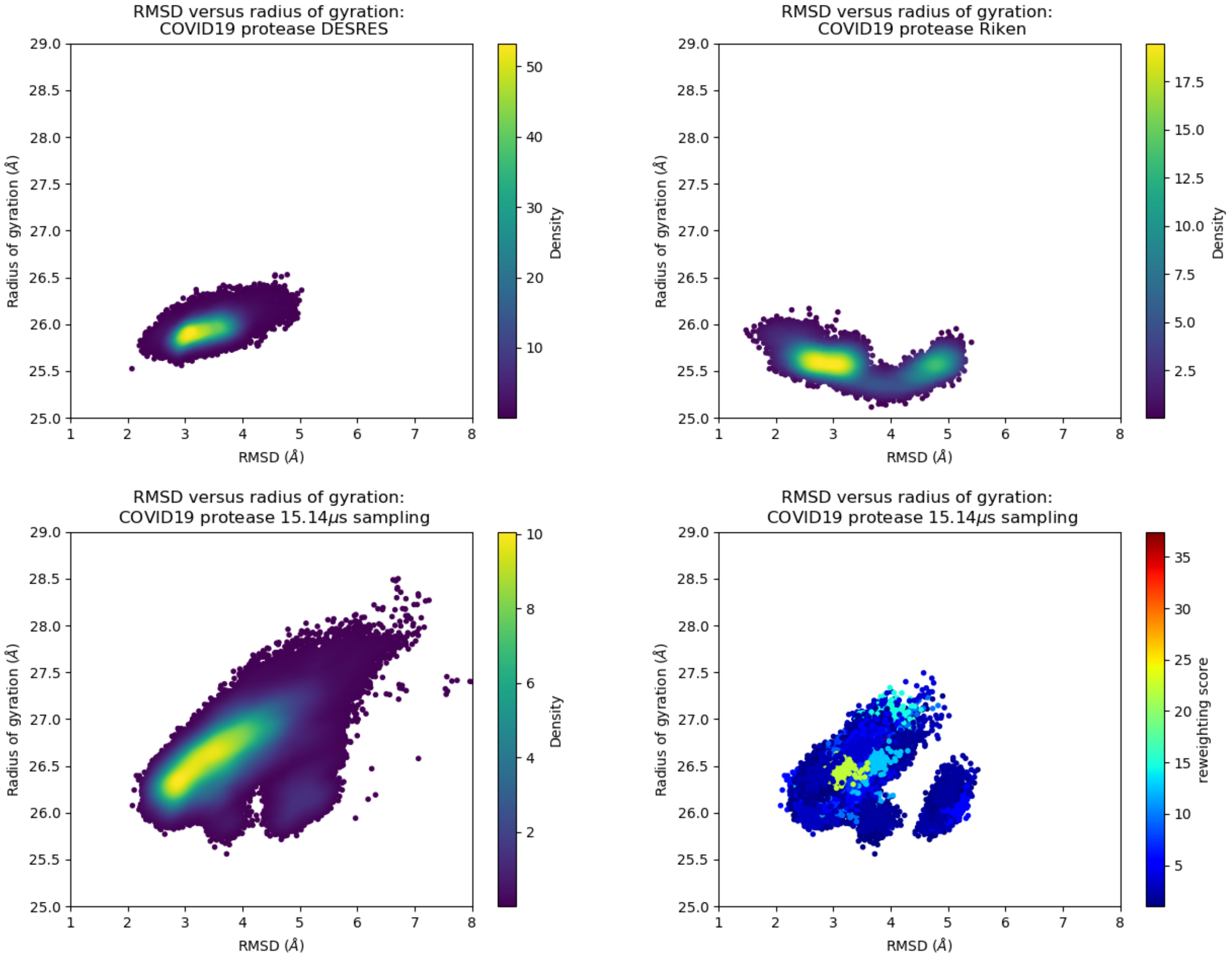

Figure 5. Root mean square deviation (RMSD) of protein backbone atoms versus radius of gyration () of DESRES **a)**, Riken **b)** datasets and AMOEBA 15.14μs **c)**. In figure **d)**, we colored the 15.14μs simulation according to their reweighting score. We only show structure which have a score larger than 1 which correspond to very likely probable states. Moreover, this reduce the dataset from 151400 structures to 33100, emphasizing the large free energy surface exploration of our simulation. Such Figure can be compared directly to the RIKEN one to evaluate the effect of the different force fields (see text)

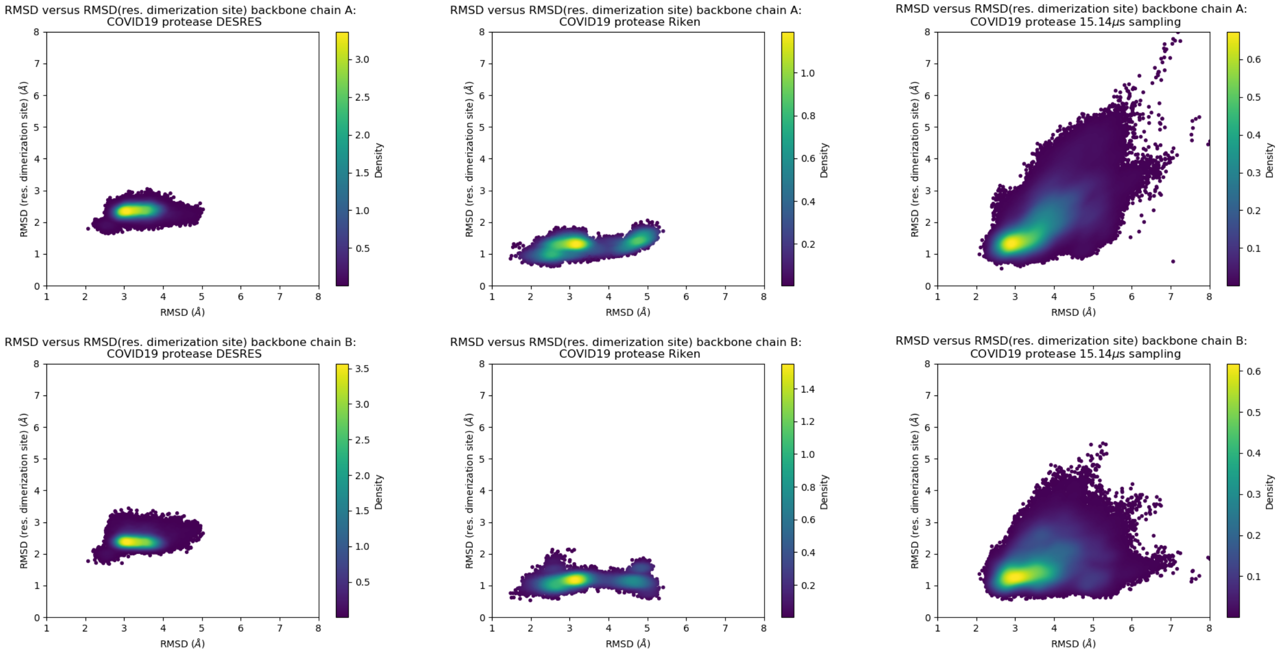

Figure 6. Root mean square deviation (RMSD) of protein backbone atoms versus RMSD of chain A and B dimerization site backbone atoms of DESRES **a)** **d)**, Riken **b)** **e)** datasets and AMOEBA 15.14μs **c)** **f)**. The dimerization site residus for chain A and B being: Arg4, Ser10, Gly11, Glu14, Asn28, Ser139, Phe140, Ser147, Glu290, Arg298.

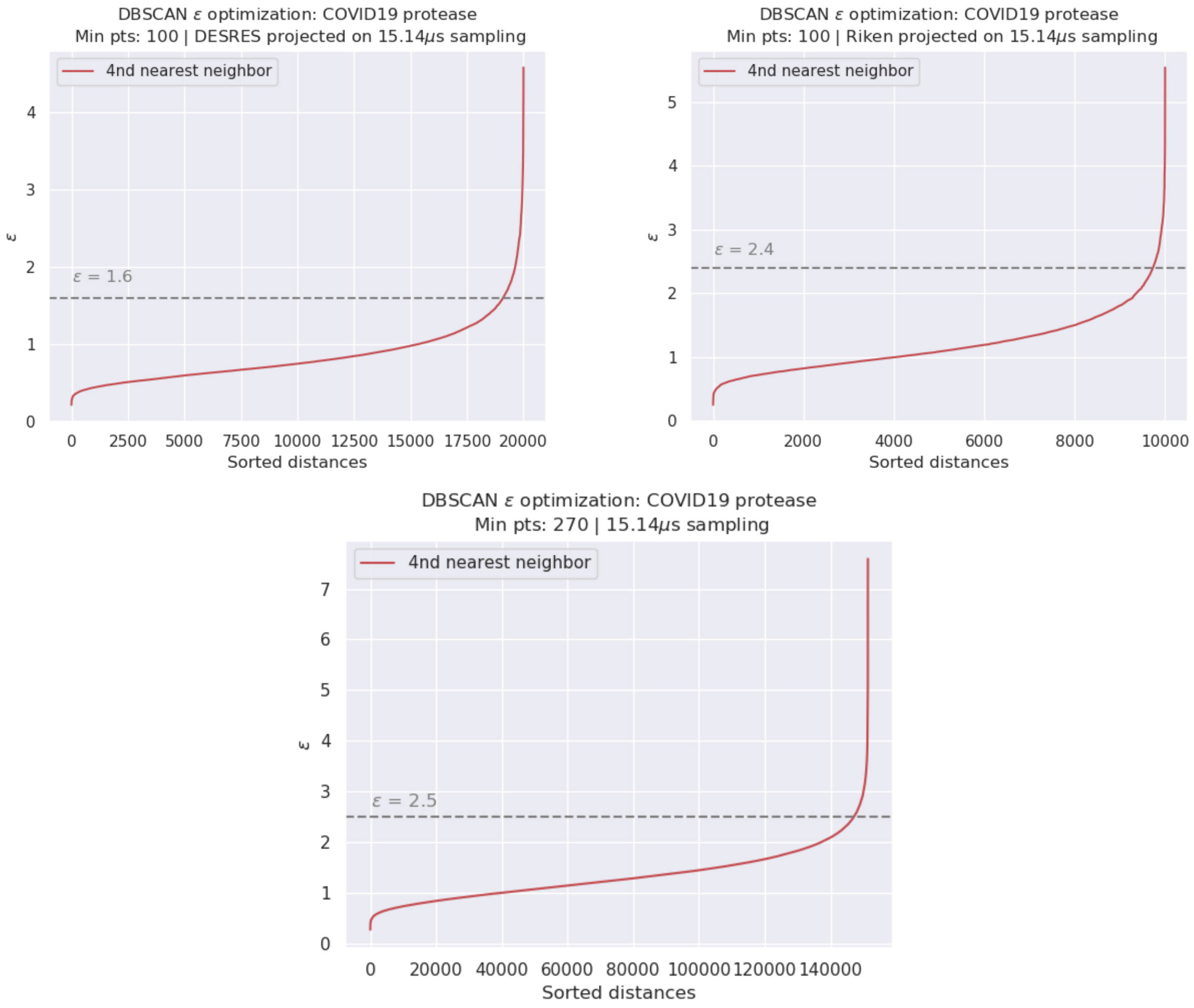

Figure 7. DBSCAN parameterization of **a)** DESRES (100μs), evaluated every 4 frames, **b)** Riken (10μs) datasets and **c)** tinker-HP 15.14μs simulation with the 4-nearest neighbor graph procedure.

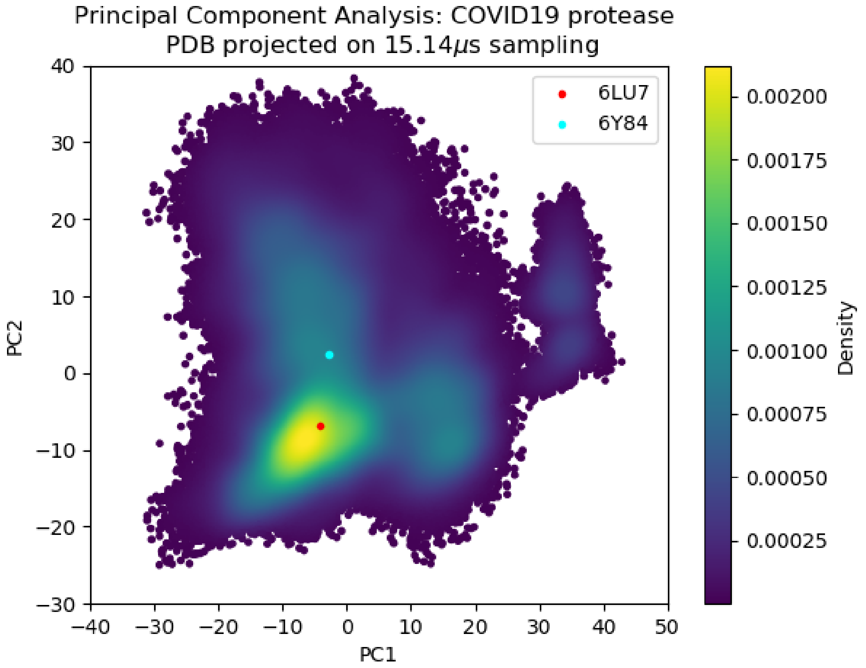

Figure 8. Projection of 6LU7 and 6Y82 pdb on the first two PCA components fitted to 15.14μ of the simulation.

Protomer 1) **Protomer 2)**

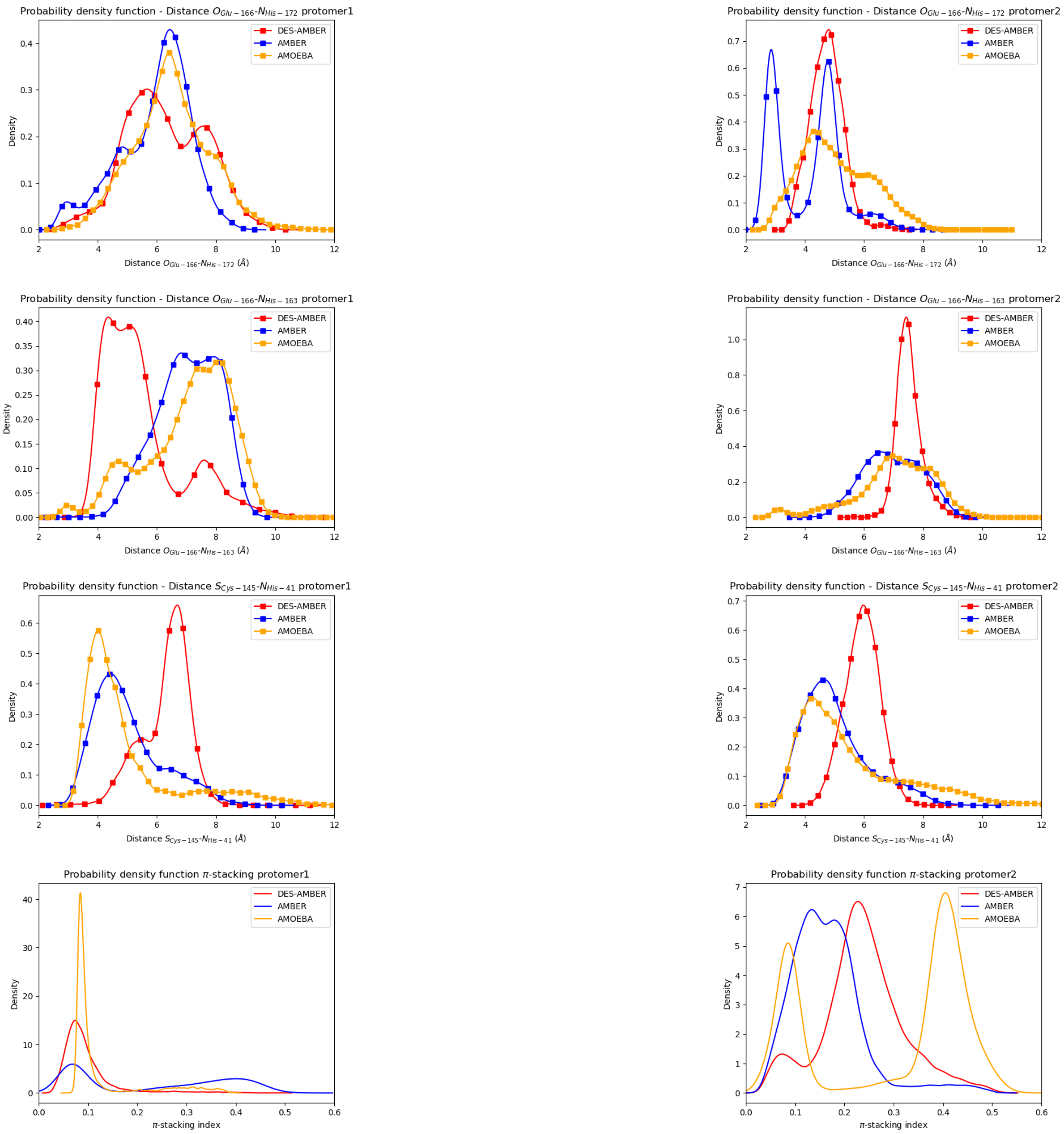

Figure 9. Representation of several interactions of interest of Glu166 (with His163 and 172), within the catalytic dial and for the π–π stacking between Phe140 and His163. Each calculations have been performed on the both chains of the system for each simulations. All simulations performed at pH=7.4 (neutral histidine residues)

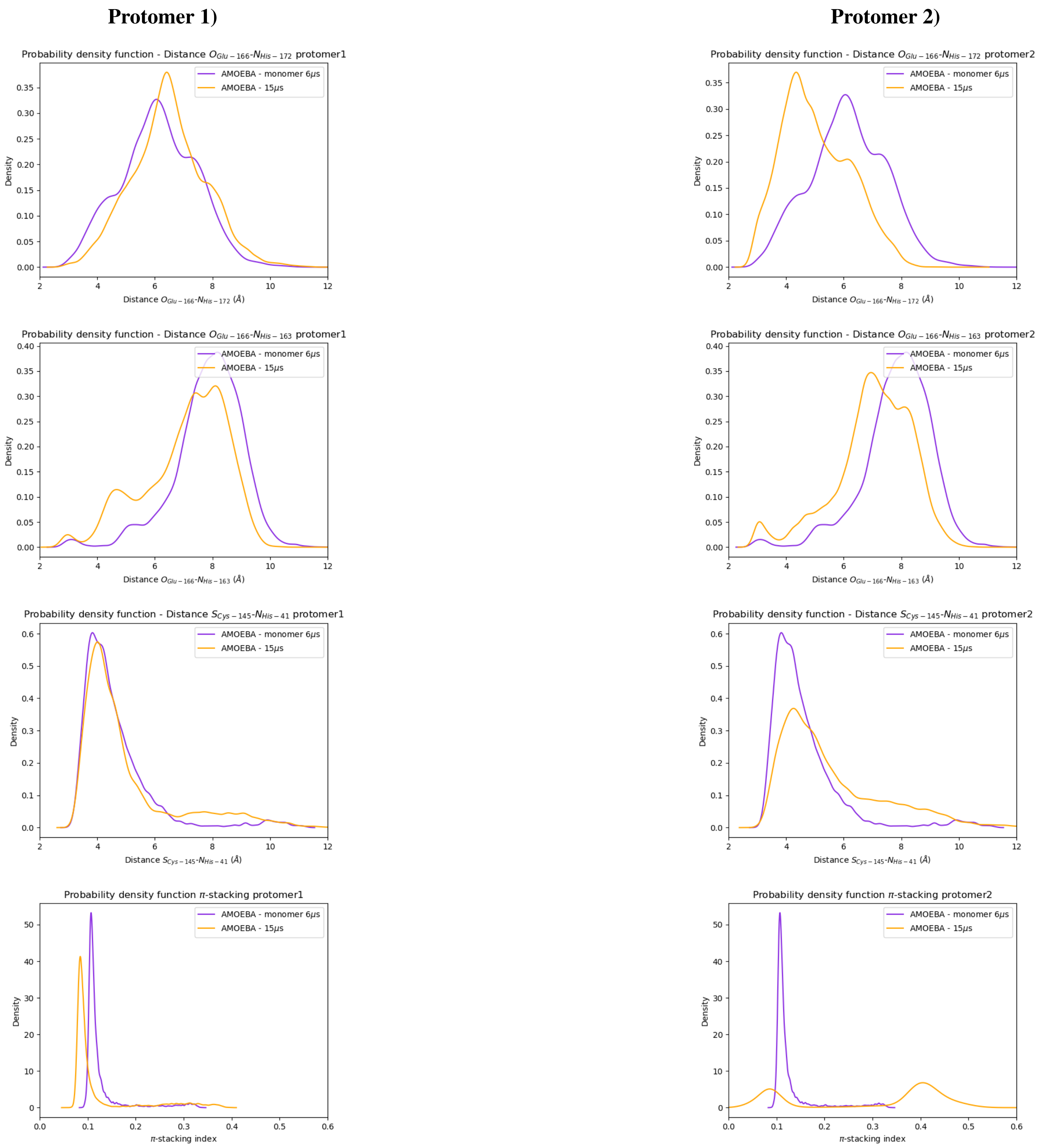

Figure 10. Representation of several interactions of interest of Glu166 (with His 163 and 172), within the catalytic dial and for the π–π stacking between Phe140 and His163. Each calculations have been performed on the both chains of the system for the 15.14μs dimer simulation and compared with the 6μs simulation of the isolated monomer (all simulations were performed at pH=7.4 with neutral histidine residues).

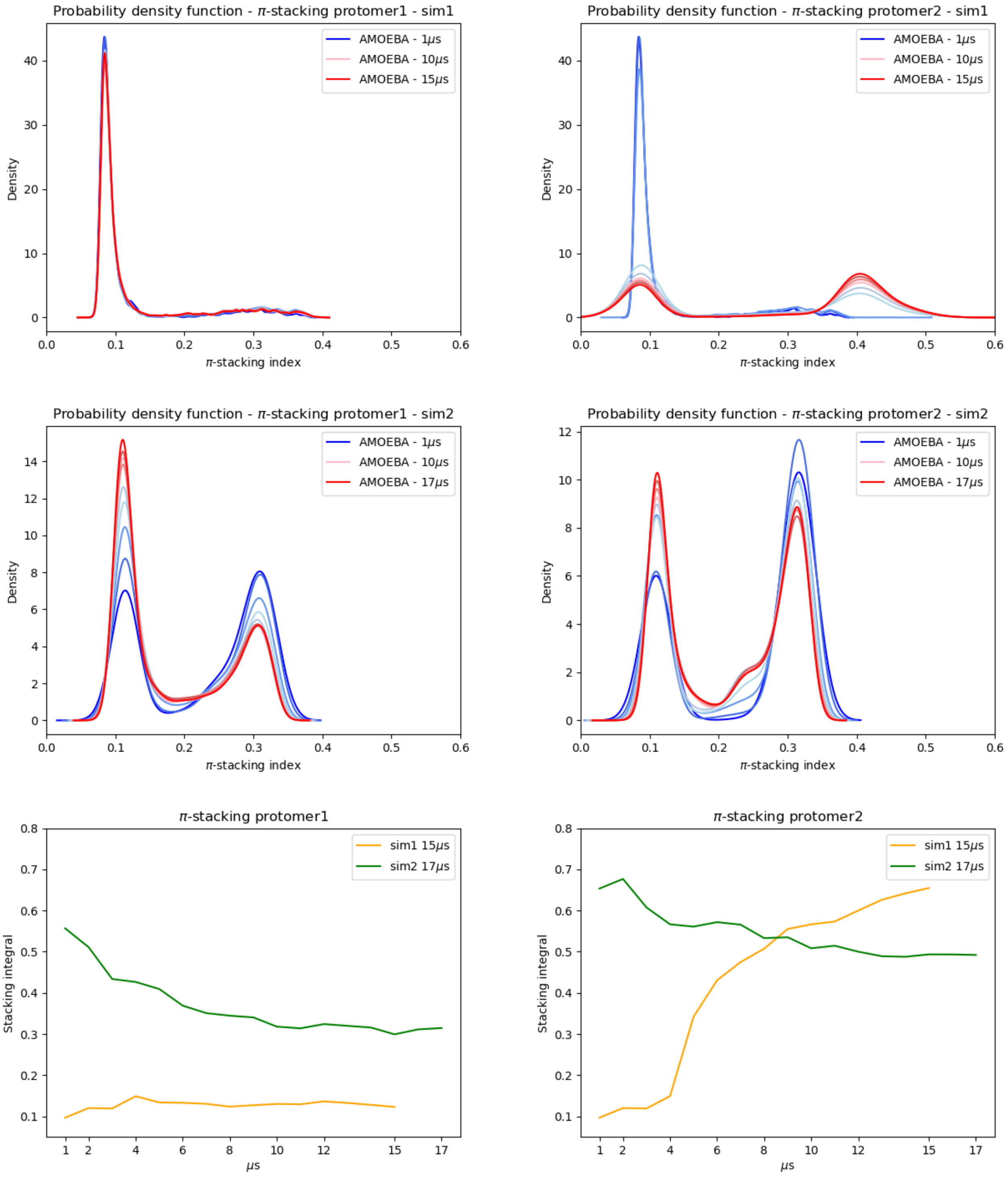

Figure 11. π-π stacking convergence for both protomers within simulation 1 (pH=7.4, neutral histidines) and simulation 2 (pH=6, protonated His172 for both protomers) **a)**, **b)** and **c)**, **d)**. Integral of the probability density function of the π-π stacking (chose superior at 0.25) over the simulation time for both protomers. **e)** and **f)**

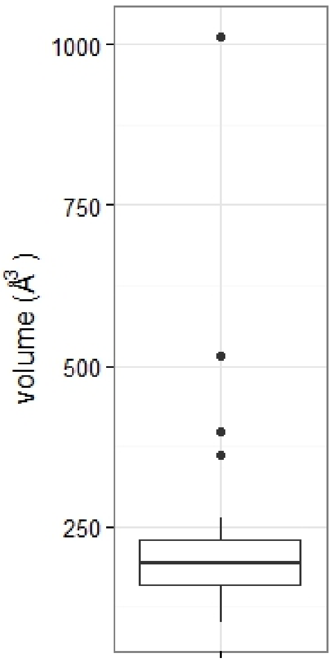

Figure 12. Boxplot of the distribution of volumes (automatically computed with DoGSite Scorer, in [3]) among the pockets corresponding to the 'T_c4_s6_P9' pocket. (Due to difference in their algorithms, DoGSite Scorer computed volumes are different from POVME)

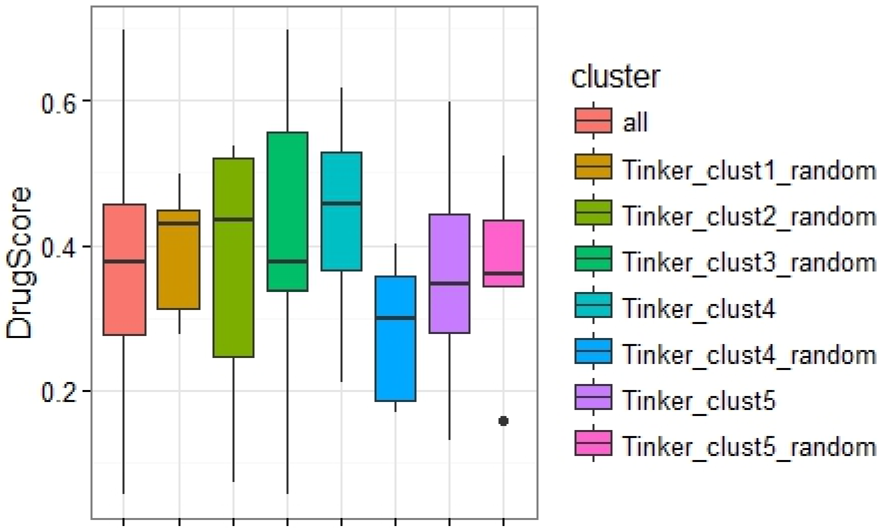

Figure 13. Boxplots of the distribution of DrugScore (automatically computed with DoGSite Scorer) among the pockets corresponding to the 'T_c4_s6_P9' pocket.

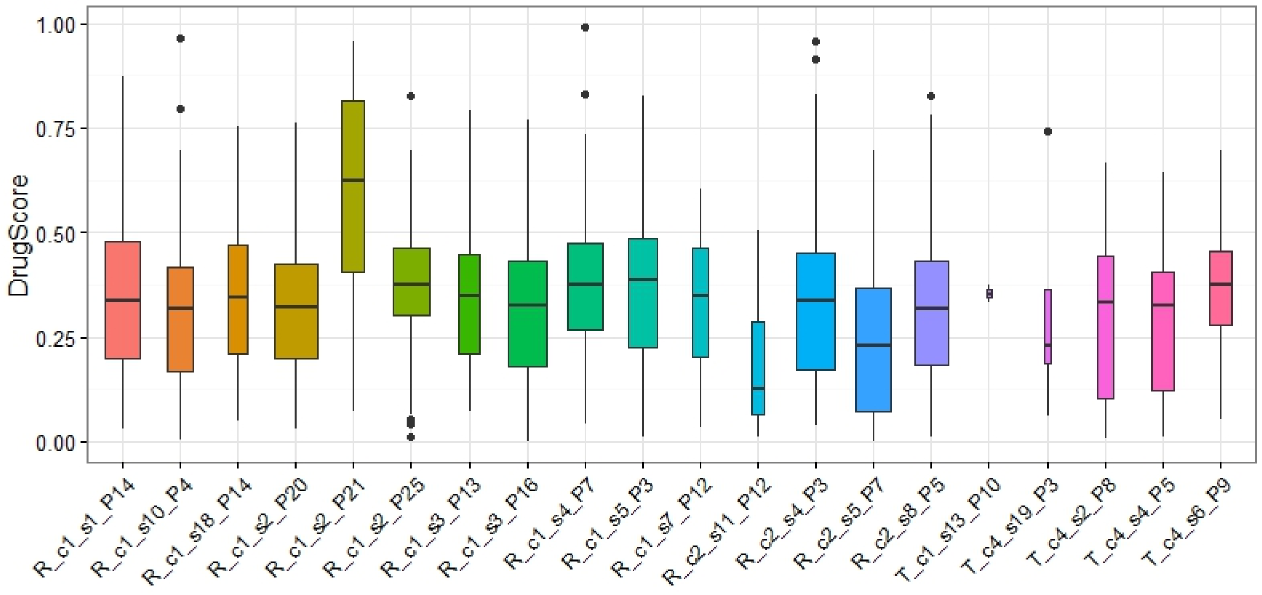

Figure 14. Boxplots of the distribution of DrugScore (automatically computed with DoGSite Scorer) for each newly identified pocket, i.e. pockets that were not detected in the 6LU7 structure.

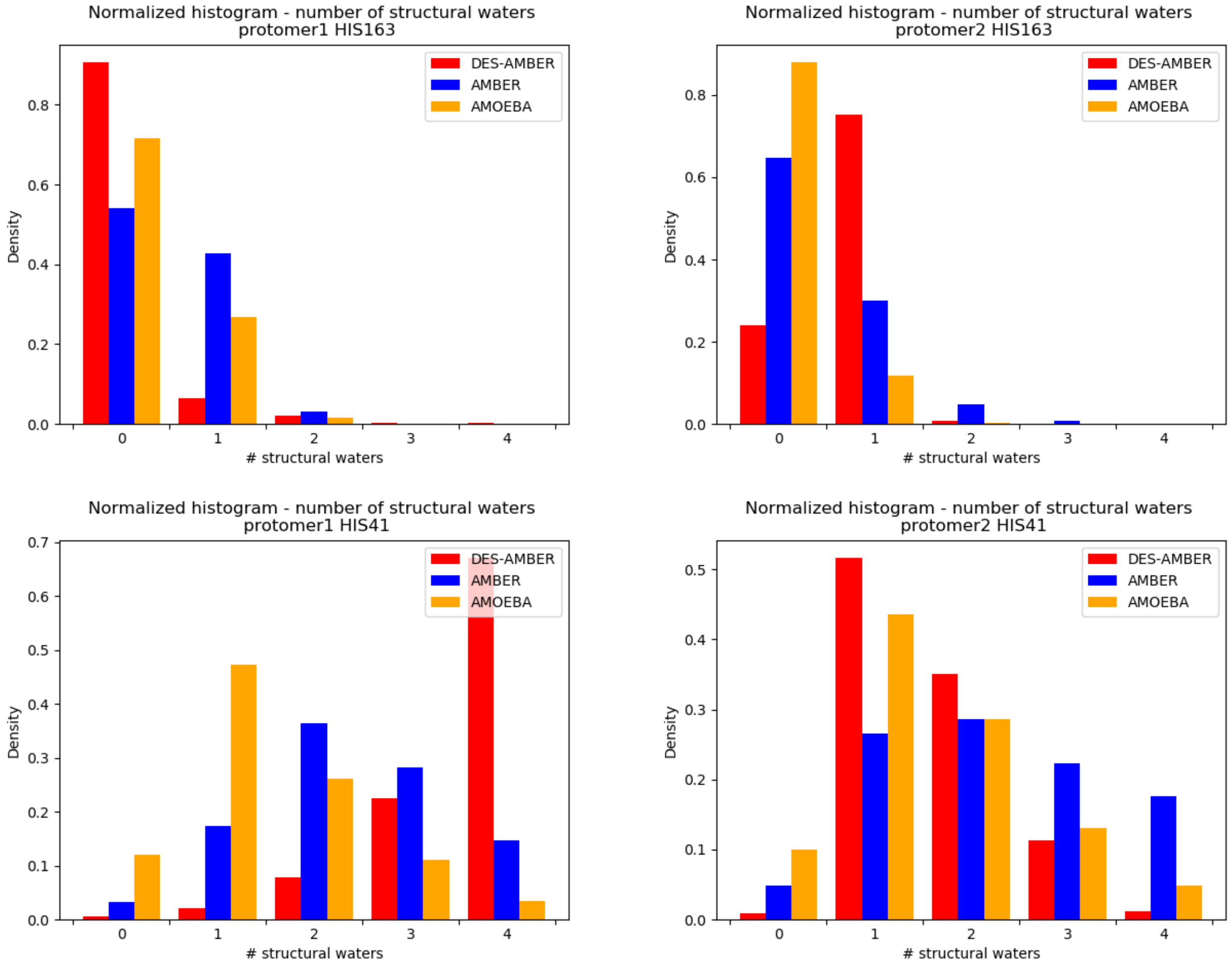

Figure 15. Number of structural water molecules around His163 **a)**, **b)** and His41 **c), for protomer 1 and 2**, **d)**.

Protomer 1) **Protomer 2)**

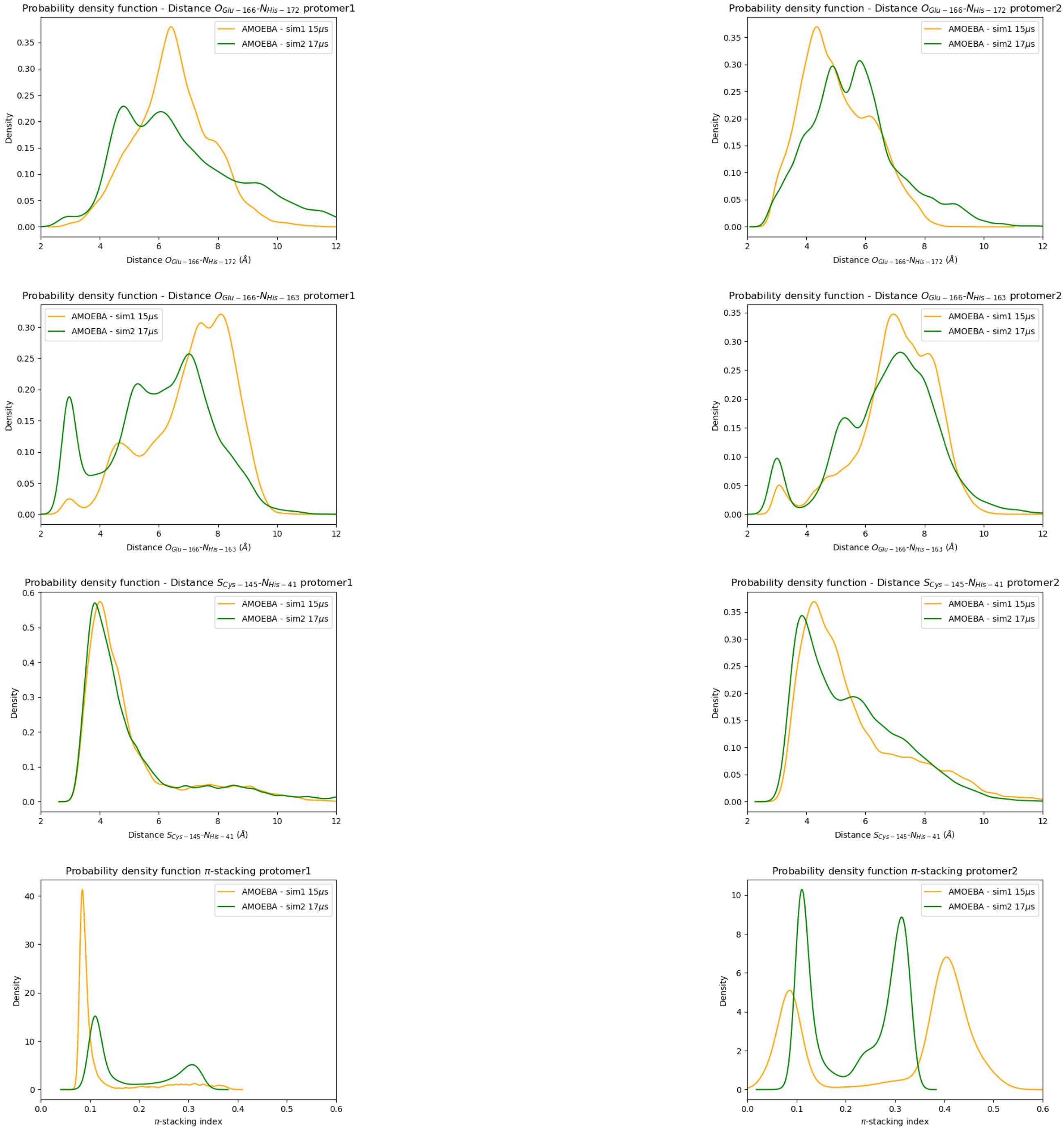

Figure 16. Representation of several interactions of interest of Glu166 (with His 163 and 172), within the catalytic dial and for the π–π stacking between Phe140 and His163 (pH 7.4 and 6).

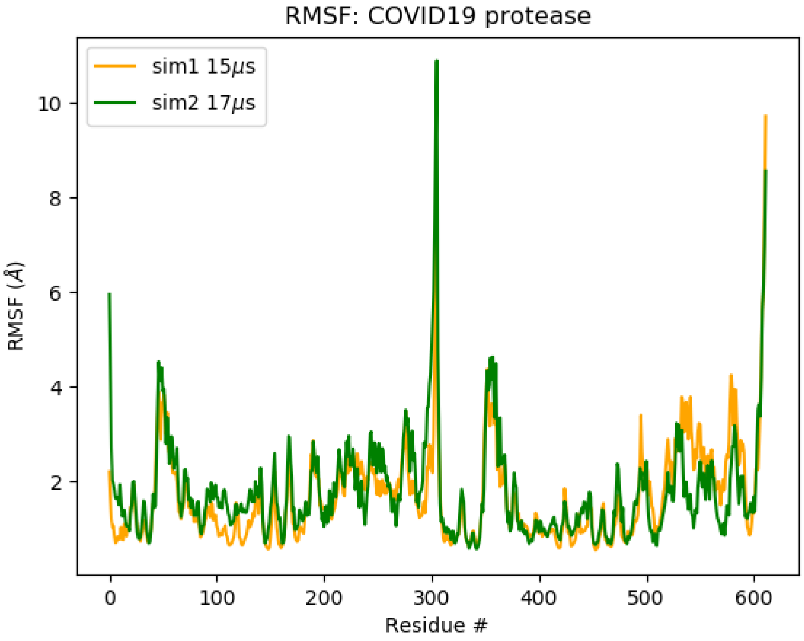

Figure 17. Root mean square fluctuation (RMSF) for pH 7.4 and pH 6.
